# Plant Disease Resistance-Related Pathways Recruit Beneficial Bacteria by Remodeling Root Exudates upon Bacillus cereus AR156 Treatment

**DOI:** 10.1128/spectrum.03611-22

**Published:** 2023-02-14

**Authors:** Bingye Yang, Mingzi Zheng, Wenpan Dong, Peiling Xu, Ying Zheng, Wei Yang, Yuming Luo, Jianhua Guo, Dongdong Niu, Yiyang Yu, Chunhao Jiang

**Affiliations:** a Department of Plant Pathology, College of Plant Protection, Nanjing Agricultural University, Nanjing, China; b Key Laboratory of Integrated Management of Crop Disease and Pests, Ministry of Education/Key Laboratory of Integrated Pest Management on Crops in East China, Ministry of Agriculture/Key Laboratory of Plant Immunity, Nanjing Agricultural University, Nanjing, China; c Engineering Center of Bioresource Pesticide in Jiangsu Province, Nanjing, China; d Jiangsu Key Laboratory for Eco-Agricultural Biotechnology around Hongze Lake/Jiangsu Collaborative Innovation Center of Regional Modern Agriculture and Environmental Protection, Huaiyin Normal University, Huai’an, China; University of Massachusetts Amherst

**Keywords:** *B. cereus* AR156, plant disease resistance-related pathway, soil microbiome, root exudates

## Abstract

The environmentally friendly biological control strategy that relies on beneficial bacterial inoculants to improve plant disease resistance is a promising strategy. Previously, it has been demonstrated that biocontrol bacteria treatments can change the plant rhizosphere microbiota but whether plant signaling pathways, especially those related to disease resistance, mediate the changes in rhizosphere microbiota has not been explored. Here, we investigated the complex interplay among biocontrol strains, plant disease resistance-related pathways, root exudates, rhizosphere microorganisms, and pathogens to further clarify the biocontrol mechanism of biocontrol bacteria by using plant signaling pathway mutants. Bacillus cereus AR156, which was previously isolated from forest soil by our laboratory, can significantly control tomato bacterial wilt disease in greenhouse and field experiments. Moreover, compared with the control treatment, the B. cereus AR156 treatment had a significant effect on the soil microbiome and recruited 35 genera of bacteria to enrich the rhizosphere of tomato. Among them, the relative rhizosphere abundance of nine genera, including *Ammoniphilus*, *Bacillus*, *Bosea*, *Candidimonas*, *Flexivirga*, *Brevundimonas*, *Bordetella*, *Dyella*, and *Candidatus_Berkiella*, was regulated by plant disease resistance-related signaling pathways and B. cereus AR156. Linear correlation analysis showed that the relative abundances of six genera in the rhizosphere were significantly negatively correlated with pathogen colonization in roots. These rhizosphere bacteria were affected by plant root exudates that are regulated by signaling pathways.

**IMPORTANCE** Our data suggest that B. cereus AR156 can promote the enrichment of beneficial microorganisms in the plant rhizosphere by regulating salicylic acid (SA) and jasmonic acid (JA)/ethylene (ET) signaling pathways in plants, thereby playing a role in controlling bacterial wilt disease. Meanwhile, Spearman correlation analysis showed that the relative abundances of these beneficial bacteria were correlated with the secretion of root exudates. Our study reveals a new mechanism for SA and JA/ET signals to participate in the adjustment of plant resistance whereby the signaling pathways adjust the rhizosphere microecology by changing the root exudates and thus change plant resistance. On the other hand, biocontrol strains can utilize this mechanism to recruit beneficial bacteria by activating disease resistance-related signaling pathways to confine the infection and spread of pathogens. Finally, our data also provide a new idea for the in-depth study of biocontrol mechanisms.

## INTRODUCTION

Tomato bacterial wilt is a soilborne disease caused by Ralstonia solanacearum, which seriously restricts the quality and yield of tomatoes and causes considerable economic losses worldwide (1, 2). The control strategies of bacterial wilt mainly include breeding-resistant varieties and physical and chemical control measures. However, due to the wide host range, diverse genotypes, and obvious physiological and pathogenic differentiation of R. solanacearum, conventional agricultural strategies cannot effectively control the disease over long periods (3). In recent years, biological control of bacterial wilt by biocontrol microorganisms has attracted extensive attention. Studies have proven that Pseudomonas, *Bacillus*, *Streptomyces*, *Trichoderma*, and other biocontrol microorganisms can be effective at controlling bacterial wilt (4–8). Bacillus cereus AR156 is a plant growth-promoting rhizobacterium, originally isolated from forest tree rhizosphere soil in our laboratory, which can prevent and control a wide variety of plant diseases. On the one hand, biocontrol microorganisms can directly inhibit the growth and reproduction of pathogens through antibacterial or bacteriolytic activity (9, 10). On the other hand, they can indirectly inhibit pathogens by stimulating the plant’s immune system, a phenomenon called induced systemic resistance (ISR) (11, 12). ISR is generally regulated by interconnected signaling pathways in which plant hormones, especially ethylene (ET), salicylic acid (SA), and jasmonic acid (JA), play important regulatory roles (13–15).

SA and JA are members of the innate immune system and play an important role in the immune defense response of higher plants (16). SA is known to be effective in defending against biotrophic pathogens by activating the expression of genes related to systemic acquired resistance and defense, while JA and ET mainly activate the expression of wound response-related genes involved in plant responses to necrotrophic pathogens and herbivorous insects (17–19). These two pathways are mostly antagonistic: elevated biotroph resistance is often correlated with increased necrotroph susceptibility, and elevated necrotroph resistance is often correlated with enhanced susceptibility to biotrophs (20). Increasing the content of JA in plants is beneficial to the infection of various pathogens. Some plant pathogens, including R. solanacearum, have developed toxins and effectors to inhibit host SA signaling by activating the JA pathway to infect plants (21). When plants are infected by pathogens, plants inhibit JA signaling and activate SA signaling through different mechanisms to enhance defense against pathogens (16, 22). Plant mutants with reduced SA content (heterotopic expression of the bacterial NahG gene and dysfunction of the SA biosynthesis gene SID2/ICS1) showed reduced local and systemic resistance and were more susceptible to infection by biotrophic pathogens, while exogenous application of SA restored resistance (23).

Recent studies have shown that biocontrol microorganisms can also inhibit the occurrence of bacterial wilt by remodeling the structure and function of rhizosphere soil microbial communities (24, 25). Increasing evidence shows that the rhizosphere soil microbiota is related to the occurrence of soilborne diseases (26–28). Beneficial soil microbial communities can protect plants from various diseases, such as potato scab caused by *Streptomyces scabies* (29), tomato wilt caused by Fusarium oxysporum (30), and damping-off disease of the sugar beet caused by Rhizoctonia solani (31). Exogenous addition of a large number of biocontrol bacteria can change the community composition, species richness, and symbiotic network correlation of plant rhizosphere microbiota and inhibit the occurrence of soilborne diseases by promoting the enrichment of beneficial microorganisms or inhibiting the accumulation of potential pathogens in the rhizosphere (25, 28, 32). In addition to increasing plant morbidity, pathogens enriched in the plant rhizosphere also change the composition of rhizosphere microbial communities and reduce the diversity of rhizosphere bacteria and the correlation of symbiotic networks (33, 34). Therefore, analyzing the complex interactions among biocontrol bacteria, pathogens, and rhizosphere microbiota is one of the keys to understanding how biocontrol bacteria improve plant disease resistance by reshaping the rhizosphere soil microbial community.

Root exudates are a variety of biochemical substances actively or passively secreted by plant roots and not only serve as carbon or nitrogen sources to provide nutrients and energy to soil microorganisms but can also serve as signal substances to recruit or inhibit specific microbial groups, which are key factors involved in regulating the assembly of rhizosphere microbial communities (35, 36). Root exudates mainly include soluble sugars, amino acids, organic acids, fatty acids, and secondary metabolites, forming a diverse chemical environment (37). The composition of root exudates is not a uniform or static property and varies depending on plant species, developmental stage, root traits, environmental conditions, nutrition, and soil type, among other factors (38, 39). Many components of plant root exudates have been proven to be involved in regulating the formation of rhizosphere microbial communities as microbial sensing signals (40). For example, certain phenolic compounds released from tobacco roots can recruit Agrobacterium tumefaciens C58 (41); l-malic acid secreted from roots of *Arabidopsis* can selectively recruit Bacillus subtilis FB17 to colonize at the rhizosphere, activating systemic acquired resistance, thereby protecting the uninfected part from Pseudomonas syringae infection (42); legumes may exude specific metabolites into their surrounding environment to optimize the bacterial population for successful symbiosis with the rhizobiome (43); and certain components (e.g., glutamic acid and alanine) exuded from roots influence the microbial composition of the cotton rhizosphere to determine the disease status of plants in monocropped soils (44). Therefore, it is of great significance to explore the rhizosphere soil microbiota and its relationship with environmental factors for disease control.

The biocontrol strain B. cereus AR156 can enhance plant resistance to pathogens through the SA and JA/ET signaling pathways (45). It has been proven that the SA and JA/ET signaling pathways not only regulate plant disease resistance but also can change the amount of plant root exudates and the composition of rhizosphere microorganisms. For example, the secretion of defense proteins in the roots of *npr1-1* mutant plants and the NahG transgenic line with SA damage in *Arabidopsis* is lower than that in wild-type plants (46). The secretion of asparagine, ornithine, and tryptophan in the roots of two JA/ET pathway mutants of *Arabidopsis* is less than that in the wild type (47). The SA signaling pathway is involved in the formation of the rhizosphere microbial community and modulates colonization of the root by specific bacterial families (48). The SA and JA/ET signaling pathways can change plant root exudates, which are some of the most important drivers shaping plant rhizosphere microbial communities. Therefore, we hypothesized that B. cereus AR156 could modulate plant root exudates through disease resistance signaling pathways, such as SA and JA/ET, thereby enriching rhizosphere microbiota in the soil that are beneficial to plant health.

To test this hypothesis, 15 groups of soil data under different conditions were collected for 16S amplicon sequencing, and the root exudates of different tomato lines were collected for nontargeted metabolomics analysis in this study. We analyzed the differences in rhizosphere microbiota of different tomato lines under the biocontrol bacteria or pathogens treatment and their correlation with root exudates. This study reveals a new mechanism via which SA and JA/ET signals participate in the regulation of plant resistance and provides a new idea for the study of biocontrol mechanisms.

## RESULTS

### B. cereus AR156 can effectively control tomato bacterial wilt disease.

**FIG 1:**
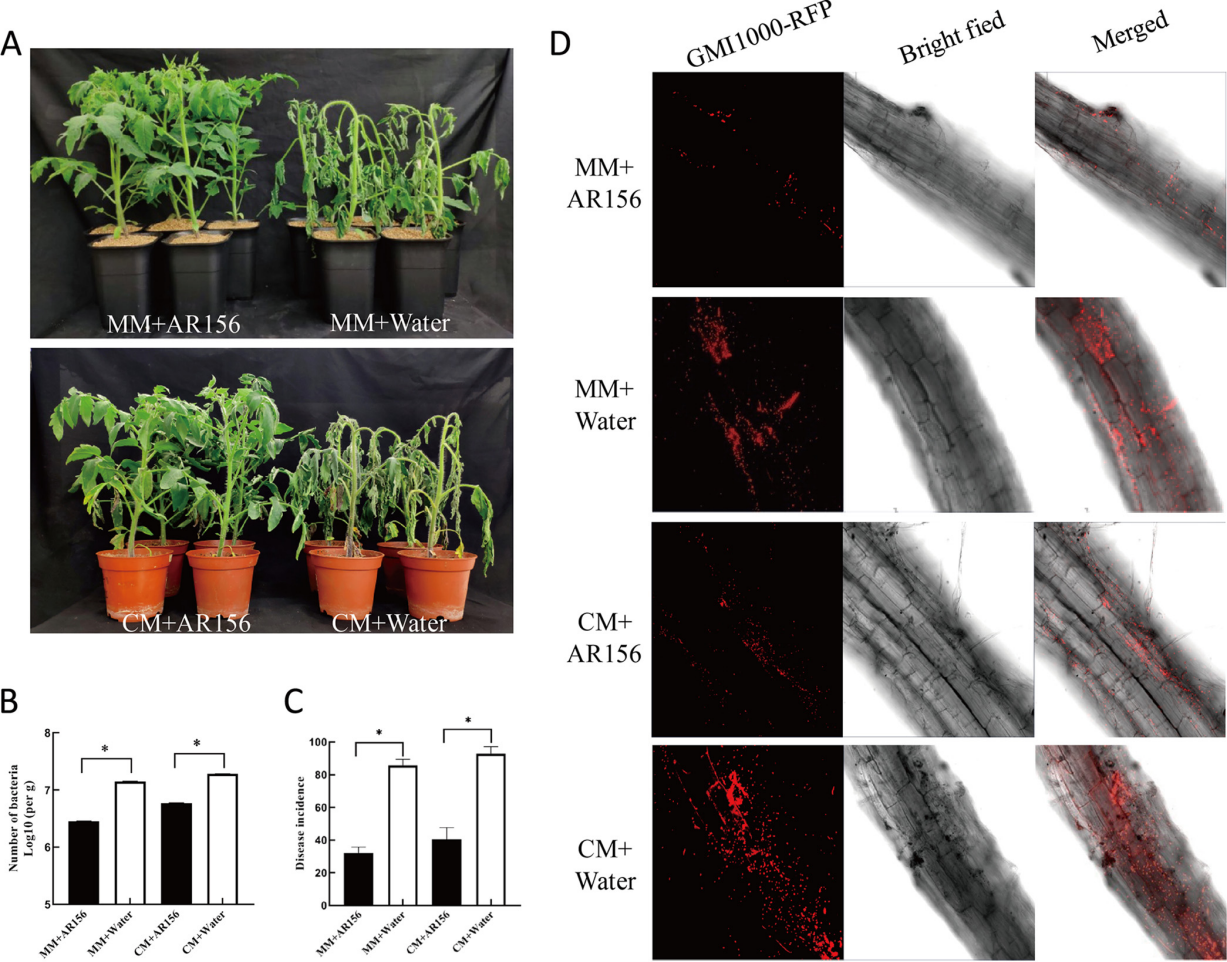
B. cereus AR156 can significantly control and reduce disease severity caused by R. solanacearum GMI1000. Plants were pretreated with B. cereus AR156 or sterile water as a mock control. Five days later, roots were irrigated with R. solanacearum GMI1000. (A) The symptoms of bacterial wilt disease development on tomato seedlings 10 days after R. solanacearum GMI1000 inoculation in each treatment in the greenhouse experiment. The experiment was repeated 3 times, and 12 seedlings were treated each time. (B) Bacteria were quantified in extracts of roots by serial dilution at day 7 after inoculation. The asterisk indicates statistically significant differences as determined with Duncan’s new multiple range test (*P* < 0.05) between treatments. The experiment was repeated 3 times, and 6 seedlings were treated each time. Bars represent the average of 3 replicates, and error bars show standard deviations. (C) Incidence statistics of tomato plants with different treatments after inoculation with R. solanacearum GMI1000 for 10 days. *, *P *< 0.05, statistically significant differences as determined with Duncan’s new multiple range test between treatments. The experiment was repeated 3 times, and 24 seedlings were treated each time. Bars represent the average of three replicates and error bars show standard deviations. (D) The colonization of GMI1000-RFP at the roots of tomatoes under different treatments was observed by laser confocal microscopy. The experiment was repeated 3 times, and 6 seedlings were treated each time.

Preliminary research from our laboratory found that B. cereus AR156 can effectively control tomato bacterial wilt. The results of the field test showed that the biocontrol efficacy of B. cereus AR156 against tomato bacterial wilt reached 70%, and the yield per Mu was approximately 2,150 kg, which was 1,300 kg higher than that of the control group (Fig. S1 in the supplemental material). We investigated the biocontrol efficacy of B. cereus AR156 against bacterial wilt on two wild tomato varieties (MM and CM) in a greenhouse. The results showed that B. cereus AR156 could reduce the disease severity of two tomato varieties and significantly improve the resistance of plants to bacterial wilt (Fig. 1A). Seven days after inoculation with R. solanacearum GMI1000, the titers of bacteria in the roots of the plants were detected. The results of colony plate counting showed that the titers of pathogens in the roots of tomato seedlings treated with B. cereus AR156 were significantly less than those in tomato seedlings treated with sterile water at *P *< 0.05 (Fig. 1B). We computed the incidence rate of tomato seedlings after 10 days of inoculation with R. solanacearum, and the results showed that the incidence of bacterial wilt of tomato seedlings treated with B. cereus AR156 was significantly lower than that of tomato seedlings treated with water (Fig. 1C). In further experiments, the R. solanacearum GMI1000 strain with a red fluorescent protein (RFP) fluorescent label was used to inoculate tomato seedlings with different treatments to observe the colonization of R. solanacearum in plant roots, and the observation results from laser confocal microscopy were consistent with the results of the pathogenicity test and incidence measurement (Fig. 1D). In summary, the results indicated that B. cereus AR156 could effectively control tomato bacterial wilt disease.

### B. cereus AR156 affects the composition and abundance of bacterial communities in plant rhizosphere soil.

**FIG 2:**
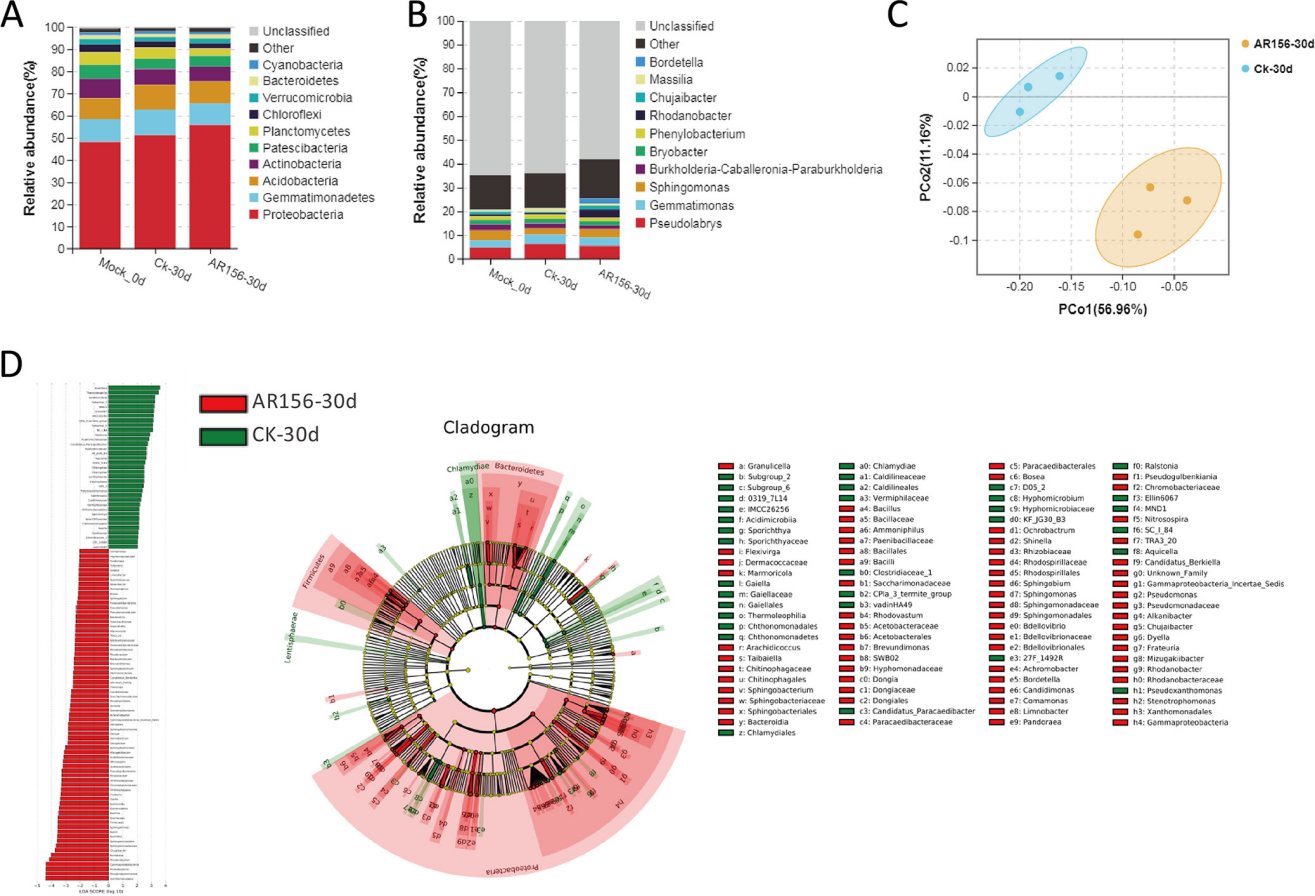
General descriptions of B. cereus AR156-treated and untreated soil bacterial communities. (A) The relative abundance (%) of the major phyla present in the bacterial communities in different treatments. (B) The relative abundance (%) of the major genus present in the bacterial communities in different treatments. (C) The principal coordinate (PC) analysis with Bray-Curtis distances on the taxonomic profile (at the OTU level) for AR156-treated and untreated rhizosphere soil bacteria. (D) Differential species based on linear discriminant effect analysis (LDA >2). The ring radiating from inside to outside represents the taxonomic level of phylum, class, order, family, and genus. Each small circle at different taxonomic levels represents a species at that taxonomic level. The diameter of the small circle is directly proportional to the relative abundance.

Rhizosphere soil microorganisms are very important for plants, and these microorganisms can improve plant tolerance to biotic and abiotic stresses. To understand the effect of B. cereus AR156 treatment on bacterial communities in rhizosphere soil, the rhizosphere soil of the tomato after different treatments was collected for 16S amplicon sequencing. After B. cereus AR156 treatment, at the phylum level, the majority of actual sequence variants belonged to *Proteobacteria* (55.7%), *Gemmatimonadetes* (9.79%), *Acidobacteria* (9.77%), *Chloroflexi* (16.1%), and *Actinobacteria* (6.75%) after B. cereus AR156 treatment (Fig. 2A). At the genus level, the majority of actual sequence variants belonged to *Pseudolabrys* (5.50%), *Gemmatimonas* (3.61%), *Sphingomonas* (3.41%), *Rhodanobacter* (3.4%), and *Bordetella* (2.38%) (Fig. 2B).

To explore the effect of B. cereus AR156 on rhizosphere microbiota, we compared the differences in the root microbiota of tomato plants treated with sterile water and B. cereus AR156. The results of principal coordinate analysis (PCoA) based on Bray-Curtis distances showed that the water-treated samples (CK-30d) and AR156-treated samples (AR156-30d) were clearly clustered into two distinct groups, indicating that B. cereus AR156 had an affect on the rhizosphere soil bacterial community composition (Fig. 2C). To illustrate the specific affected species, linear discriminant analysis effect size (LEfSe) analysis was performed. The results showed that compared with the samples treated with sterile water, B. cereus AR156 treatment significantly increased the relative abundances of *Proteobacteria* (linear discriminant effect analysis [LDA] score = 4.40), *Firmicutes* (LDA score = 3.55), and *Bacteroidetes* (LDA score = 3.46) at the phylum level (Fig. 2D). B. cereus AR156 treatment significantly increased the relative abundances of 35 genera in rhizosphere soil, including *Rhodanobacter*, *Bordetella*, *Chujaibacter*, *Bacillus*, *Dyella*, *Frateuria*, and 29 other genera, while *Gaiella*, *Ellin6067*, *Candidatus_Paracaedibacter*, and nine other genera were significantly reduced in relative abundance (Fig. 2D and Table S1).

### Control of tomato bacterial wilt by B. cereus AR156 is related to SA and JA/ET signaling pathways.

To determine whether the biocontrol activity of B. cereus AR156 is due to a direct effect or indirect effect, we first performed a plate antagonism test, and the results showed that B. cereus AR156 cannot directly inhibit the growth of R. solanacearum GMI1000 (Fig. S2). This led us to speculate that the reason why B. cereus AR156 improves plant disease resistance may be because it induces certain disease resistance responses in plants. Subsequently, we determined the relative expression of genes related to SA and JA/ET signaling pathways in plants after B. cereus AR156 treatment, and the results showed that the expression of SA-related genes was upregulated after B. cereus AR156 treatment, while the expression of JA/ET-related genes was downregulated first and then upregulated (Fig. S3). Finally, we investigated the biocontrol efficacy of B. cereus AR156 against bacterial wilt disease on NahG transgenic lines and *Def* deletion mutants, as well as their corresponding wild-type plants MM and CM, in a greenhouse experiment. We found that the biocontrol efficacy of B. cereus AR156 on NahG transgenic lines and *Def* deletion mutants was significantly different from that on wild-type plants (Fig. S4A). The statistical analysis results showed that the control effect of B. cereus AR156 on *Def* mutants was always higher than that of wild-type CM and the control effect on NahG transgenic lines was always lower than that of wild-type MM by approximately 20% (Fig. S4B and S4C). All these results suggest that the control of tomato bacterial wilt by B. cereus AR156 is related to the SA and JA/ET signaling pathways.

### The SA and JA/ET signaling pathways are involved in the control of bacterial wilt disease.

To further clarify whether plant resistance to tomato bacterial wilt is related to the SA and JA/ET signaling pathways, we investigated the sensitivity of NahG transgenic lines and *Def* deletion mutants, as well as their corresponding wild-type plants MM and CM to R. solanacearum. We consistently observed stronger symptom development in NahG transgenic lines than in their corresponding wild-type plants, and the *Def* mutant had higher resistance against R. solanacearum GMI1000 infection than the wild-type CM (Fig. S5A). Seven days after inoculation with Ralstonia solanacearum GMI1000, the titers of bacteria in the roots of plants were determined. The results of colony plate counting showed that the titers of pathogens in the roots of NahG transgenic lines were significantly higher than those in wild-type MM at *P *< 0.05, and the titers of pathogens in the roots of *Def* deletion mutants were significantly less than those in wild-type CM at *P *< 0.05 (Fig. S5B). The results of scanning electron microscopy observation of the browning site of roots showed that the bacterial content observed in MM roots was less than that in NahG transgenic lines and the bacterial content observed in CM roots was more than that in *Def* mutants (Fig. S5C). The colonization of the GMI1000 strain with an RFP fluorescent label in the plant roots was observed by laser confocal microscopy and was consistent with the detection results of titer (Fig. S5D). In summary, the findings indicated that the ability of the tomato to resist bacterial wilt is related to the SA and JA/ET signaling pathways.

### B. cereus AR156 induces rhizosphere bacterial community changes similar to the effects of disease resistance signaling pathways.

**FIG 3:**
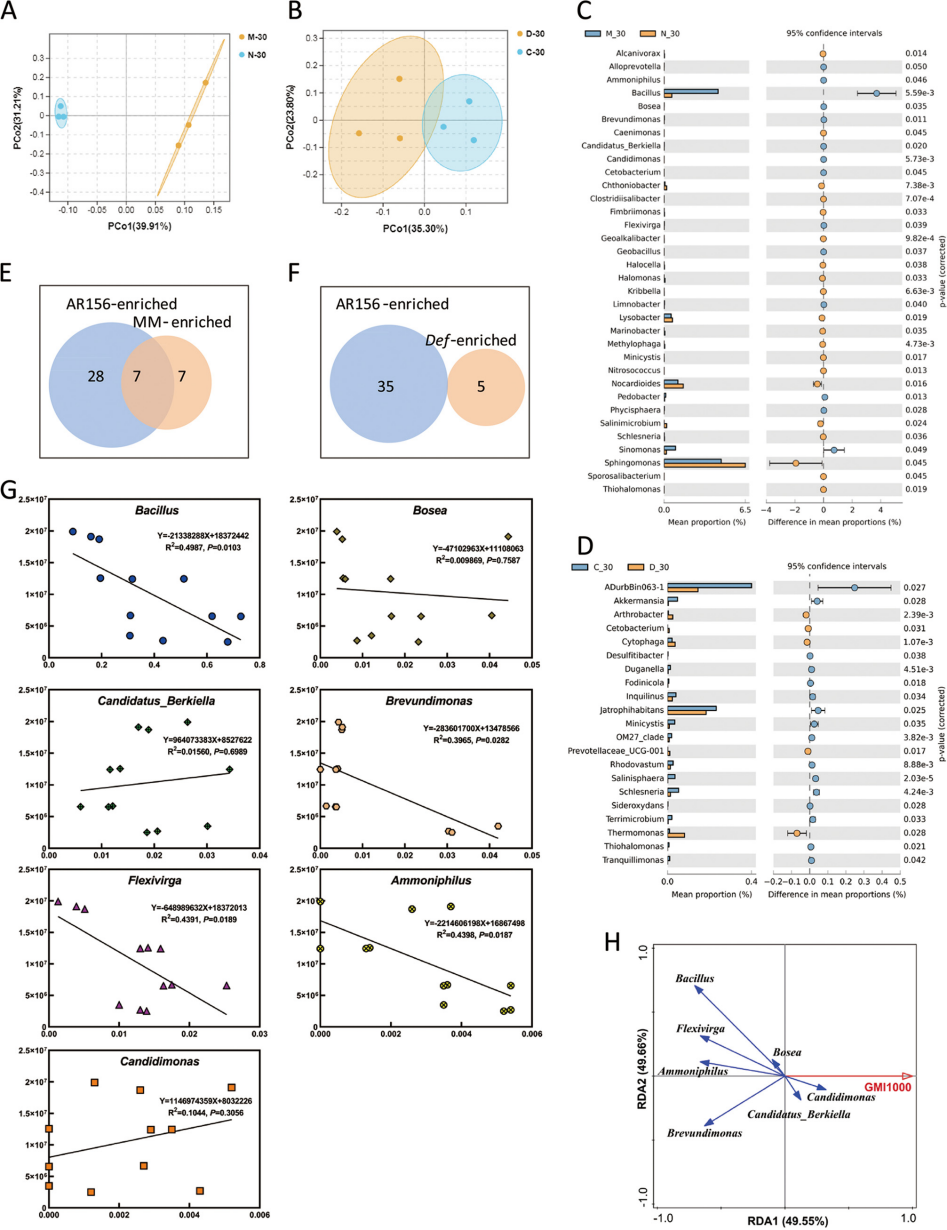
Changes in the rhizosphere microbiota caused by disease resistance-related pathways and their relationship with B. cereus AR156-induced changes in the rhizosphere microbiota. (A) Principal coordinate analysis with Bray-Curtis distances on the taxonomic profile (at the OTU level) of the rhizosphere soil bacteria for wild-type MM and the transgenic line NahG. (B) Principal coordinate analysis with Bray-Curtis distances on the taxonomic profile (at the OTU level) of the rhizosphere soil bacteria for wild-type CM and the mutant *Def*. (C) Difference in relative abundance between rhizosphere samples of wild-type MM and NahG transgenic tomato at the genus level (Welch’s *t* test; *P *< 0.05). (D) Difference in relative abundance between rhizosphere samples of wild-type CM and *Def* deletion mutants at the genus level (Welch’s *t* test; *P *< 0.05). (E) Venn diagram of shared and unique genus numbers observed in AR156-enriched and MM-enriched soil bacterial communities. (F) Venn diagram of shared and unique genus numbers observed in AR156-enriched and *Def*-enriched soil bacterial communities. (G) The relationship between targeted bacterial groups and the colonization of R. solanacearum. The *x* axis indicates the relative abundance of targeted bacterial groups (%), and the *y* axis indicates the colonization of R. solanacearum. (H) RDA diagram of targeted bacterial groups and the colonization of R. solanacearum at the genus level.

Previous experiments showed that the resistance of the NahG transgenic line to R. solanacearum GMI1000 was weaker than that of the wild-type plant MM (Fig. S5). We compared the differences in the rhizosphere bacterial community of wild-type plant MM and NahG transgenic line 30 days after planting. The PCoA based on Bray-Curtis distances showed that the rhizosphere soil samples of wild-type MM and NahG transgenic plants can be significantly separated along the PCo1 axis, indicating that overexpression of the *NahG* gene has a great impact on the rhizosphere soil bacterial community composition (Fig. 3A). We performed LEFse analysis at the phylum level on rhizosphere soil microorganisms of wild-type MM and the NahG transgenic line grown for 30 days. The results of LEfSe analysis showed that the bacteria enriched in the rhizosphere of wild-type MM were mainly concentrated in *Firmicutes* and *Cyanobacteria*, and the bacteria enriched in the rhizosphere of NahG transgenic plants were mainly concentrated in *Verrucomicrobia* and *Armatimonadetes* (Fig. S6A). To clearly display the differences at the genus level, Welch’s *t* test was performed using STAMP software. The results showed that a total of 34 genera were significantly different between the 2 groups of samples, of which 14 genera were enriched in the rhizosphere of wild-type MM and 20 genera were enriched in the rhizosphere of NahG transgenic line (Fig. 3C). A comparison of 14 genera enriched in the wild-type MM rhizosphere with 35 genera enriched by B. cereus AR156 treatment found that seven of them were present in both sets, namely, *Ammoniphilus*, *Bacillus*, *Bosea*, *Candidimonas*, *Flexivirga*, *Brevundimonas*, and *Candidatus_Berkiella* (Fig. 3E). Linear correlation and redundancy analyses between the relative abundances of these seven genera in the rhizosphere soil and the colonization level of R. solanacearum in plant roots were performed. The results showed that the relative abundances of *Bacillus*, *Brevundimonas*, *Flexivirga*, and *Ammoniphilus* in rhizosphere soil were significantly negatively correlated with the colonization level of R. solanacearum in plant roots, and the relative abundance of *Bosea* in rhizosphere soil was also negatively correlated with the colonization level of R. solanacearum in plant roots, but the correlation was not significant (Fig. 3G and H). Therefore, *Bacillus*, *Brevundimonas*, *Flexivirga*, and *Ammoniphilus* are the key genera induced and enriched through the SA signaling pathway by B. cereus AR156 and play an important role in controlling bacterial wilt disease. Although *Bosea*, *Candidimonas*, and *Candidatus_Berkiella* can also be enriched through the B. cereus AR156-induced SA pathway, they are not related to the control of bacterial wilt disease.

Similarly, we compared the differences in rhizosphere bacterial community of wild-type plant CM and the *Def* mutant 30 days after planting. The result of PCoA based on Bray-Curtis distances showed that the rhizosphere soil samples of wild-type CM and *Def* mutant plants could not be significantly separated along the PCo1 or PCo2 axis, indicating that deletion of the *def-1* gene has a limited effect on the rhizosphere soil bacterial community composition (Fig. 3B). We performed LEFse analysis at the phylum level on rhizosphere soil microorganisms of wild-type CM and *Def* mutant plants grown for 30 days. The results of LEfSe analysis showed that the bacteria enriched in the rhizosphere of wild-type CM were mainly concentrated in *Planctomycetes*, *Verrucomicrobia*, *Armatimonadetes*, and *Halanaerobiaeota* (Fig. S6B). To clearly display the differences at the genus level in rhizosphere soil microorganisms, Welch’s *t* test was performed using STAMP software. The results showed that there were significant differences in 21 genera between the two groups of samples, including 16 genera enriched in the rhizosphere of wild-type CM and 5 genera enriched in the rhizosphere of *Def* mutants (Fig. 3D). Comparing the five genera enriched in the *Def* mutant plant rhizosphere with 35 genera enriched by B. cereus AR156 treatment, no shared genera were found (Fig. 3F).

### B. cereus AR156 indirectly induces rhizosphere bacterial community changes through disease resistance signaling pathways and consequently controls bacterial wilt.

**FIG 4:**
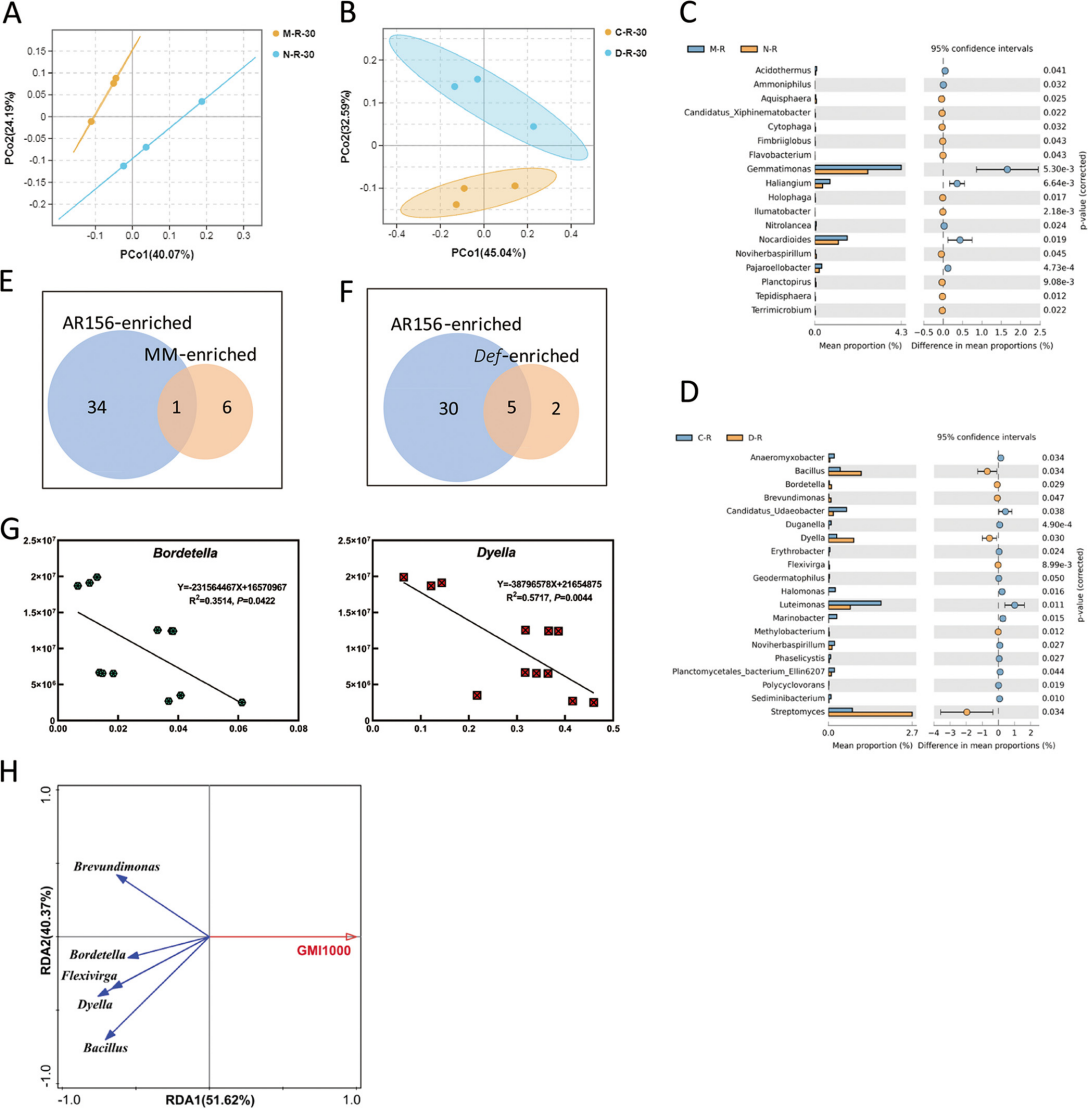
Changes in the rhizosphere microbiota caused by disease resistance-related pathways and their relationship with B. cereus AR156-induced changes in the rhizosphere microbiota when threatened by pathogens. (A) Principal coordinate analysis with Bray-Curtis distances on the taxonomic profile (at the OTU level) of the rhizosphere soil bacteria for wild-type MM and the transgenic line NahG after inoculation with R. solanacearum. (B) Principal coordinate analysis with Bray-Curtis distances on the taxonomic profile (at the OTU level) of the rhizosphere soil bacteria for wild-type CM and the mutant line *Def* after inoculation with R. solanacearum. (C) Difference in relative abundance between rhizosphere samples of wild-type MM and NahG transgenic line at the genus level after inoculation with R. solanacearum (Welch’s *t* test; *P *< 0.05). (D) Difference in relative abundance between rhizosphere samples of wild-type CM and *Def* deletion mutants at the genus level after inoculation with R. solanacearum (Welch’s *t* test; *P *< 0.05). (E) Venn diagram of shared and unique genus numbers observed in AR156-enriched and MM-enriched soil bacterial communities after inoculation with R. solanacearum. (F) Venn diagram of shared and unique genus numbers observed in AR156-enriched and *Def*-enriched soil bacterial communities after inoculation with R. solanacearum. (G) The relationship between targeted bacterial groups and the colonization of R. solanacearum. The *x* axis indicates the relative abundance of targeted bacterial groups (%), and the *y* axis indicates the colonization of R. solanacearum*. Bacillus*, *Brevundimonas* and *flexivirga* are not shown because they have already been shown in Fig. 3. (H) RDA diagram of targeted bacterial groups and the colonization of R. solanacearum at the genus level.

We compared the differences in the rhizosphere bacterial community of wild-type plant MM and transgenic line NahG after inoculation with R. solanacearum. The results of PCoA based on Bray-Curtis distances showed that the rhizosphere soil samples inoculated with R. solanacearum of wild-type MM and transgenic line NahG could not be significantly separated along the PCo1 or PCo2 axis (Fig. 4A). To clearly display the differences at the genus level in rhizosphere soil microorganisms between these two groups, Welch's *t* test was performed using STAMP software. The results showed that a total of 18 genera were significantly different between the two groups of samples, of which seven genera were enriched in the rhizosphere of wild-type MM after inoculation with R. solanacearum GMI1000 and 11 genera were enriched in the rhizosphere of NahG transgenic line after inoculation with R. solanacearum GMI1000 (Fig. 4C). To explore the relationship between the B. cereus AR156-enriched bacteria and the SA or JA/ET signaling pathways during R. solanacearum infection, these 7 genera enriched in the wild-type MM rhizosphere were compared with the 35 genera enriched by B. cereus AR156, and only *Ammoniphilus* was present in both groups (Fig. 4E).

Similarly, we compared the differences in the rhizosphere bacterial community of wild-type plant CM and mutant line *Def* after inoculation with R. solanacearum. The result of PCoA based on Bray-Curtis distances showed that the rhizosphere soil samples inoculated with R. solanacearum of wild-type CM and the mutant line *Def* can be significantly separated along the PCo2 axis, indicating that the rhizosphere bacterial community composition and abundance of wild-type CM and the mutant line *Def* are quite different during the resistance of R. solanacearum infection (Fig. 4B). To clearly display the differences at the genus level in rhizosphere soil microorganisms between these two groups, Welch's *t* test was performed using STAMP software. The results showed that there were significant differences in 20 genera between the 2 groups of samples, including 13 genera enriched in the rhizosphere of wild-type CM after inoculation with R. solanacearum GMI1000 and 7 genera enriched in the rhizosphere of *Def* mutants after inoculation with R. solanacearum GMI1000 (Fig. 4D). The seven genera enriched in the mutant line *Def* rhizosphere were compared with the 35 genera enriched by B. cereus AR156 treatment, and it was found that 5 genera existed in both groups, namely, *Bacillus*, *Bordetella*, *Flexivirga*, *Brevundimonas*, and *Dyella* (Fig. 4F). Linear correlation and redundancy analyses between the relative abundances of these five genera in the rhizosphere soil and the colonization levels of R. solanacearum in plant roots were performed. The results showed that the relative abundances of all these five genera in rhizosphere soil were significantly negatively correlated with the colonization level of R. solanacearum in plant roots (Fig. 4G and H). Among them, the results of *Bacillus*, *Brevundimonas*, and *Flexivirga* were the same as those in Fig. 3G, so they are not shown in Fig. 4G. Therefore, we consider that these five genera are the key genera induced and enriched through the JA/ET signaling pathway by B. cereus AR156 and play an important role in controlling bacterial wilt disease.

### Root exudates are related to changes in rhizosphere bacterial community composition.

Root exudates are the main media for information exchange and energy transmission between plant roots and soil and can induce positive or negative interactions between plants and rhizosphere microbiota (49). We measured the root exudates of NahG transgenic lines, *Def* mutant line, and the MM and CM wild-type plants hydroponically cultivated for 15 days and detected 7,881 and 2,396 compounds in positive and negative ion modes. Upon applying the criterion fold change ≥2 or ≤0.5, there were a total of 3,526 differential exudates identified between the wild-type MM and NahG transgenic lines, of which 1,872 exudates were upregulated and 1654 exudates were downregulated (Table S2). There were 3,929 differential exudates between the wild-type CM and *Def* mutant lines, of which 1,855 exudates were upregulated and 2,074 exudates were downregulated (Table S3).

**FIG 5:**
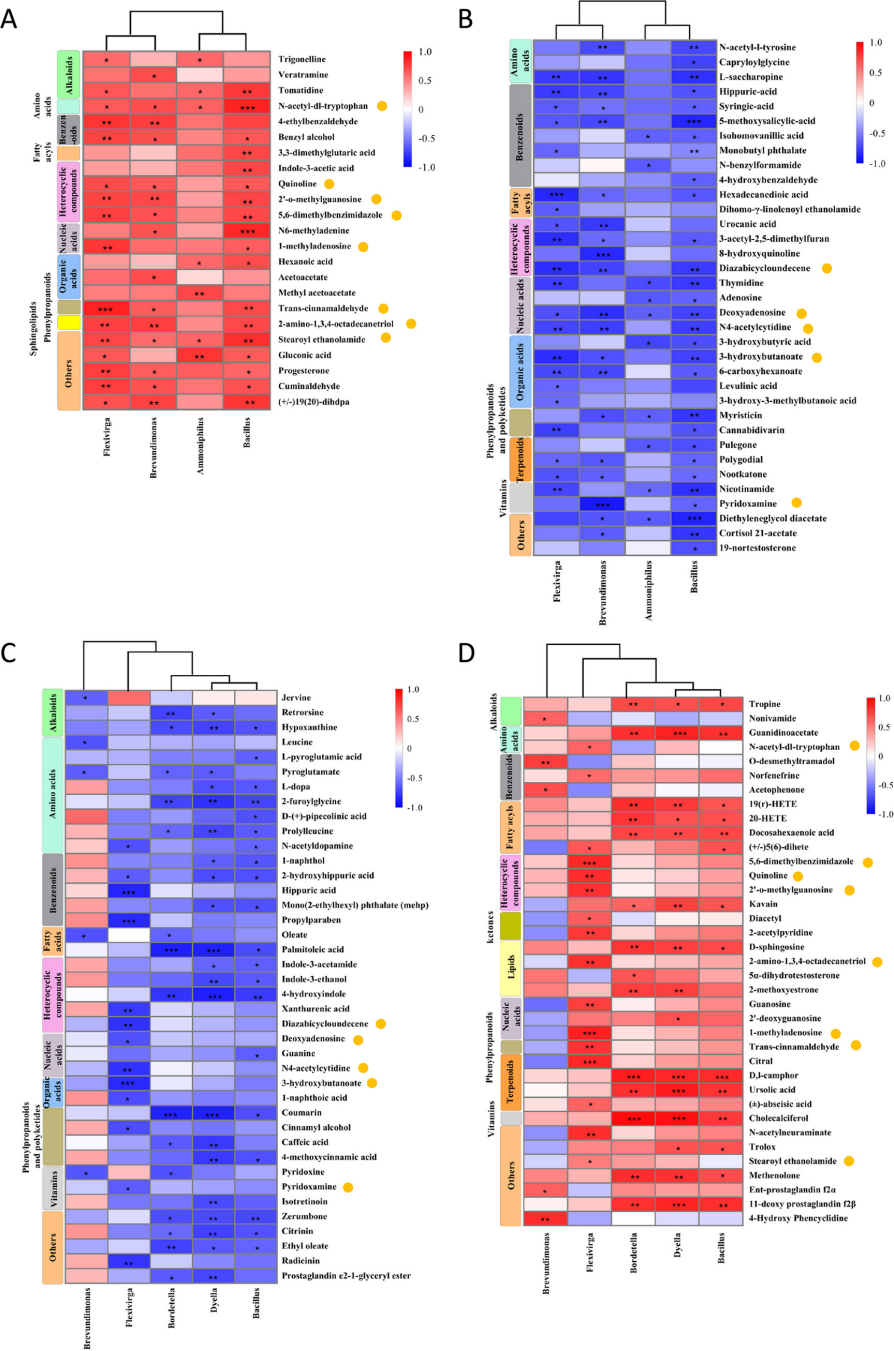
Correlation between root exudates and several rhizosphere microorganisms. (A) Heatmap analysis of the correlation between the upregulated differential exudates in wild-type MM and the NahG transgenic line and the relative abundances of four rhizosphere microorganisms. (B) Heatmap analysis of the correlation between the downregulated differential exudates in wild-type MM and the NahG transgenic line and the relative abundances of four rhizosphere microorganisms. (C) Heatmap analysis of the correlation between the upregulated differential exudates in wild-type CM and the *Def* mutant line and the relative abundances of five rhizosphere microorganisms. (D) Heatmap analysis of the correlation between the downregulated differential exudates in wild-type CM and the *Def* mutant line and the relative abundances of five rhizosphere microorganisms. The horizontal axis represents the genus name of microorganisms in soil, the vertical axis represents differential exudates, the shade of color represents the strength of the correlation, and yellow circles indicate differential exudates coexisting in the MM/NahG and CM/*Def* systems. *, *P* < 0.05; **, *P* < 0.01; ***, *P* < 0.001.

Among all the differential exudates of the wild-type MM and NahG transgenic lines, the compounds with the top three confidence levels were selected, and the data detected repeatedly in positive and negative ion modes were removed. There were 123 remaining compounds. Spearman correlation analysis was performed between the secreted amounts of these 123 differential exudates, and the relative abundances of the four key genera are analyzed in Fig. 3 (Spearman’s rank correlation coefficients and *P* values are shown in Table S4 and Table S5). Figure 5A shows the correlation between the upregulated differential exudates in wild-type MM and NahG transgenic line and the relative abundances of four rhizosphere microorganisms. Due to the large number of exudates with significant differences, not all of them are shown in the figure. The differential exudates shown in Fig. 5A were significantly correlated with at least one rhizosphere microorganism and are also shown in Fig. 5B to D. Figure 5B shows the correlation between the downregulated differential exudates in wild-type MM and NahG transgenic lines and the relative abundances of four rhizosphere microorganisms. As can be seen from Fig. 5B, several detected alkaloids were significantly positively correlated with the four rhizosphere microorganisms, while several detected terpenoids and vitamins were significantly negatively correlated with the four rhizosphere microorganisms (Fig. 5A and B). Both positively and negatively correlated exudates were present in the remaining classifications. In terms of quantity, benzenoids (9 compounds) were the most abundant in all classifications, followed by organic acids (8 compounds) and heterocyclic compounds (8 compounds). Therefore, the findings indicated that the relative abundances of *Bacillus*, *Ammoniphilus*, *Flexivirga*, and *Brevundimonas* in the rhizosphere soil were correlated with the amounts of plant root exudates.

The compounds with the top three confidence were selected from all differential exudates of wild-type CM and the *Def* mutant line, and the compounds with contradictory disease correlation were removed, retaining 114 compounds. Spearman correlation analysis was performed between the secreted amounts of these 114 differential exudates, and the relative abundances of the 5 key genera were analyzed and are shown in Fig. 4 (Spearman’s rank correlation coefficients and *P* values are shown in Table S6 and Table S7). Figure 5C shows the correlation between the upregulated differential exudates in the wild-type CM and the *Def* mutant line and the relative abundances of five rhizosphere microorganisms. Figure 5D shows the correlation between the downregulated differential exudates in the wild-type CM and the *Def* mutant line and the relative abundances of five rhizosphere microorganisms. The relative abundance of *Brevundimonas* was not significantly correlated with most of the differential exudates (Fig. 5C and D). Therefore, the relative abundance of *Brevundimonas* in rhizosphere soil is independent of the changes in exudates caused by the JA/ET signaling pathway. Several differentially secreted organic acids were all negatively correlated with the other four rhizosphere microorganisms, while several detected terpenoids were significantly positively correlated with the other four rhizosphere microorganisms (Fig. 5C and D). Both positively and negatively correlated exudates were present in the remaining classifications. In terms of quantity, the top three differentially exudated compounds were amino acids (10 compounds), heterocyclics (9 compounds), and benzenoids (8 compounds). Therefore, we speculate that compared with wild-type CM, the *Def* mutant line attracts *Bacillus*, *Bordetella*, *Brevundimonas*, *Dyella*, and *Flexivirga* to enrich them in the roots, which is related to the amounts of root exudates.

By comprehensively comparing the several subplots in Fig. 5, we found that 13 compounds were associated with both SA and JA/ET pathways. Among them, eight compounds, including three heterocyclic compounds, were positively correlated with rhizosphere microorganisms, namely, *N*-acetyl-dl-tryptophan, quinoline, 1-methyladenosine, 2′-*O*-methylguanosine, 2-amino-1,3,4-octadecanetriol, stearoyl ethanolamide, 5,6-dimethylbenzimidazole, and trans-cinnamaldehyde. The other five compounds were negatively correlated with these rhizosphere microorganisms, namely, deoxyadenosine, *N*^4^-acetylcytidine, diazabicycloundecene, pyredoxamine, and 3-hydroxybutanoate, two of which belonged to nucleic acids.

## DISCUSSION

The application of biocontrol strains is beneficial for plants to control bacterial wilt (4–8). Adding a large number of exogenous biocontrol bacteria to the soil may have an impact on the soil microecology. For example, adding Pseudomonas fluorescens 2P24 and CPF10 to the soil for growing cucumbers has inhibitory effects on *Cyanobacterium*, *Beta-proteobacterium*, and Staphylococcus but can promote the growth of *Bacillus* to a certain extent (50). Inoculation with Bacillus velezensis B63 or Pseudomonas fluorescens P142 in tomato root soil causes significant changes in the composition of the rhizosphere bacterial community, and the corresponding microbiome shifts may trigger plant defense against the R. solanacearum B3B (25). The interactions among biocontrol strains, soil microecology, and plant roots are complex, and the mechanism by which biocontrol strains regulate soil microbiota changes and resist soilborne diseases is still unclear. Therefore, it is very meaningful to study the effect of biocontrol strains on plant rhizosphere microbiota. Our study showed that the rhizosphere microbial community composition and abundance were significantly different between B. cereus AR156 treatment and sterile water treatment and the B. cereus AR156 treatment significantly increased the relative abundances of 35 genera in rhizosphere soil (Fig. 2 and Table S1). The 35 genera enriched by B. cereus AR156 included *Bacillus* and Pseudomonas, which are common genera of biocontrol bacteria (10, 12, 51, 52). For example, Bacillus velezensis FJAT-46737 can control tomato bacterial wilt, and Pseudomonas aeruginosa FG106 can antagonize pathogens of several agriculturally important plants (10, 53). The 35 genera enriched by B. cereus AR156 also included some other genera that have been proven to be related to resistance to biological or abiotic stresses, such as *Dyella*, which has been shown to be involved in the inhibition of wheat bare patch disease (54); *Arachidicoccus*, which is associated with the inhibition of tomato bacterial wilt (55); and *Achromobacter*, which improves the resistance of *Arabidopsis* seedlings to salt stress (56).

Biocontrol strains can protect plants from a variety of pathogens by activating the SA and JA/ET signaling pathways to induce systemic disease resistance (ISR). It is generally believed that the SA and JA/ET signaling pathways are antagonistic to each other during SAR, but the SA and JA/ET signaling pathways lack obvious antagonism during ISR. For example, Bacillus amyloliquefaciens FZB42 mediates the SA and JA/ET signaling pathways synergistically for resistance against *Phytophthora nicotianae* (57). The expression of genes and proteins related to both SA and JA/ET signaling pathways in cucumber was upregulated after *Trichoderma longibrachiatum* H9 treatment (58). Our study suggests that B. cereus AR156 treatment activates the SA and JA/ET signaling pathways of the tomato, which are involved in the control of bacterial wilt (Fig. S2). Although transgenic technology is relatively mature and has been successfully applied to more than 150 different plant species, the current literature on the study of transgenic plant microbiomes is relatively limited (59). Previous studies have shown that transgenic rice and switchgrass have no significant effect on the rhizosphere microbiota of plants, while the rhizosphere bacterial community of transgenic sugarcane changed with changes in plant root exudates (60–62). Our study suggests that there are significant differences in the rhizosphere microbiota of the NahG transgenic line compared with wild-type tomato (Fig. 3A). At the phylum level, the bacteria enriched in the rhizosphere of wild-type MM were mainly concentrated in *Firmicutes* and *Cyanobacteria*, and the bacteria enriched in the rhizosphere of NahG transgenic plants were mainly concentrated in *Verrucomicrobia* and *Armatimonadetes* (Fig. S6A). Previous reports have shown that the species of *Cyanobacteria* are key components in maintaining the stability of the disease-suppressing soil network (63). *Firmicutes* contain a variety of Gram-positive bacteria, especially *Bacillus*, which has been proven in many articles to control soilborne diseases (64–66). Therefore, the findings indicated that compared with the NahG transgenic line, wild-type MM had enriched more beneficial bacteria in the root soil, which increasesd the ability of plants to resist bacterial wilt disease. Greenhouse experiments also demonstrated that wild-type MM had stronger disease resistance than the transgenic line NahG (Fig. S5). The rhizosphere microbial community composition of JA signal deletion mutants of *Arabidopsis* is different from that of wild-type plants (47). Our results show that at the phylum level, the rhizosphere soil of wild-type CM plants enriched a large number of bacteria from *Planctomycetes* and *Verrucomicrobia* compared with the JA signaling mutant line *Def* (Fig. S6B). *Planctomycetes* have been proven to be positively correlated with the abundance of R. solanacearum in rhizosphere soil (67), and Verrucomicrobia has been shown to have higher levels in the initial soil of plants with high disease incidence in later stages (55). Therefore, we speculate that the reason for the occurrence of more serious disease in wild-type plants is partly due to the accumulated bacterial groups around the roots that are conducive to R. solanacearum infection.

The analysis results at the genus level showed that there were 34 differential genera between wild-type MM and the transgenic line NahG, 14 genera were enriched in the rhizosphere of wild-type MM, and 7 genera were consistent with the enrichment of B. cereus AR156, namely, *Ammoniphilus*, *Bacillus*, *Bosea*, *Candidimonas*, *Flexivirga*, *Brevundimonas*, and *Candidatus_Berkiella* (Fig. 3C and E). Therefore, we speculate that these seven genera are the key genera regulated by the SA signaling pathway mediated by B. cereus AR156. There were 20 differential genera between wild-type CM and the *Def* mutant line after inoculation with R. solanacearum, among which *Bacillus*, *Bordetella*, *Brevundimonas*, *Dyella*, and *Flexivirga* were consistent with the enrichment of B. cereus AR156 (Fig. 4D and F). Therefore, we speculate that these five genera play an important role in the response to R. solanacearum infection. Among these genera related to the disease resistance signaling pathway, *Bacillus* has been proven to have the ability to control tomato bacterial wilt disease (10, 12, 51); *Dyella* has been proven to be associated with the suppression of bare patch disease of wheat (54); *Bosea* has been shown to work with six other bacteria to inhibit the growth of Fusarium oxysporum (68); and *Brevundimonas* has been shown to have plant growth-promoting and antifungal activities (69–71). Previous studies have shown that the abundance of *Bordetella* in soil is negatively correlated with the relative abundance of the pathogen Fusarium oxysporum in soil, suggesting that it plays a role in inhibiting the enrichment of pathogens in the soil (72). In addition, the supernatant of *Ammoniphilus* fermentation broth has been proven to be resistant to Meloidogyne incognita (73). *Flexivirga*, *Candidimonas*, and *Candidatus_Berkiella* have not yet been studied in terms of plant disease control. In conclusion, our research provides new resources for the biological control of bacterial wilt disease in the future.

Exogenous addition of SA can activate the SA signaling pathway in plants, and spraying SA can increase the amount of 2-phenylethyl glucosinolate in root exudates of *Brassica rapa* subsp. (74). Spraying methyl jasmonate (MeJA) simulated the conditions that continuously activated the JA signaling pathway, with increased amounts of hyperfirin and other acyl phloroglucinols (APG) secreted by *Hypericum perforatum* roots and decreased secretion of catechin (75). Transcriptome sequencing of the roots and leaves of *Baphicacanthus cusia* after exogenous application of MeJA showed that the relative expressions of eight genes involved in tryptophan biosynthesis in leaves were downregulated after 7 days of treatment, and four genes related to tryptophan biosynthesis in roots were upregulated, suggesting that exogenous application of MeJA may affect the synthesis of tryptophan (76). Our study found that the effect of jasmonic acid on the tryptophan metabolic pathway was also reflected in the components of root exudates. Changes in root exudates of *Def* mutant lines are manifested not only as decreased secretion of jasmonic acid and increased secretion of its precursor 12-oxophytodienoic acid but also as decreased secretion of various metabolites in the auxin-related tryptophan metabolic pathway (Fig. S7). In addition, the amount of plant root exudates also varies with the host genotype (77, 78). Previous studies have shown that SA-impaired *npr1-1* mutant lines and NahG-overexpressing lines secrete fewer defense proteins from roots than wild-type plants (46). The *myc2* and *med25* mutant lines with JA damage secreted fewer amino acid metabolites, such as asparagine, ornithine, and tryptophan (47). Our study showed that deletion of the *def-1* gene affected the secretion of 114 root exudates and overexpression of the *NahG* gene affected the secretion of 123 root exudates.

Root exudates play an important role in regulating changes in the rhizosphere microbial community during plant defense responses (47). Plants manipulate rhizosphere microbial community composition by regulating root exudates to establish a disease-suppressing environment in the soil (79, 80). Alkaloids are a class of secondary metabolites produced by plants and are considered defense compounds (81). It has been reported that alkaloids can effectively inhibit the growth of more than a dozen pathogens, such as *Ustilaginoidea virens*, *Cochliobolus miyabeanus*, and Fusarium graminearum (82, 83). In addition to helping plants defend against infection by pathogenic microorganisms, alkaloids can also help plants resist attack by herbivores and disrupt the development and reproduction of insects (84–87). Tomatidine, the major steroidal alkaloid in tomatoes, has broad inhibitory activity against plant and human pathogens (88). In addition, tomatidine also plays a role in the formation of rhizosphere bacterial communities, affecting the enrichment of *Sphingomonadaceae* in the rhizosphere in a concentration-dependent manner (89). Our study suggests that the secretion of several alkaloids, which are significantly correlated with the relative abundances of several microbes used for the analysis, are all upregulated through the SA signaling pathway (Fig. 5A and B). Among them, tomatidine affected the enrichment of *Ammoniphilus*, *Bacillus*, and *Flexivirga* in the rhizosphere and was positively correlated with their abundance in the rhizosphere (Fig. 5A). Generally, terpenoids secreted by roots have strong antibacterial properties (90). Therefore, when pathogens are absent, some of them may repel plant rhizosphere microorganisms. Previous studies have shown that pulegone, polygodial, and derivatives with nootkatone have broad-spectrum antibacterial activities against a variety of microorganisms, including *Bacillus* (91–93). This is consistent with our analysis, which shows that pulegone, polygodial, and nootkatone are not conducive to the enrichment of *Bacillus* in the plant rhizosphere (Fig. 5B). Our analysis showed that the secretion of several terpenoids, which were significantly correlated with the relative abundances of several microbes used for the analysis, were downregulated through the SA signaling pathway (Fig. 5A and B). In terms of quantity of compounds, the transgenic line with SA damage regulated the abundance of *Ammoniphilus*, *Bacillus*, *Flexivirga*, and *Brevundimonas* in rhizosphere soil mainly by changing the secretion of benzenoids, organic acids, and heterocyclic compounds (Fig. 5A and B). Benzenoid compounds are dominant constituents of essential oils with demonstrated growth-inhibiting properties, and they may strongly influence bacterial colonization in the phyllosphere (94). The results of our analysis suggest that most of the benzenoid compounds altered through the SA and JA/ET signaling pathways are not conducive to the rhizosphere enrichment of several of the microbes shown in Fig. 5. Organic acids are the most common exogenous carbon source used by soil microorganisms. In plants, 3-hydroxybutanoate acts as a regulatory molecule that most likely influences the expression of genes involved in DNA methylation, thereby altering DNA methylation levels (95). This compound has a therapeutic effect on many human diseases, such as cancer or diseases of the nervous and circulatory systems (95). The principle underlying its use in the treatment of cancer is its selective activation of the genes encoding cyclins, which stops cells in the G_1_ growth phase and blocks cell division (96). Recent research showed that 3-hydroxybutanoate promoted cell growth at low concentrations and inhibited cell growth at high concentrations (97). This is similar to the results obtained by our analysis. High concentrations of 3-hydroxybutanoate are not conducive to the enrichment of *Flexivirga*, *Bacillus*, and *Brevundimonas* in the rhizosphere, while low concentrations are conducive to the enrichment of *Flexivirga*, *Bacillus*, and *Brevundimonas* in the rhizosphere.

The results of Spearman correlation analysis showed that the abundances of *Bacillus*, *Flexivirga*, *Brevundimonas*, *Bordetella*, and *Dyella* in the rhizosphere regulated by the JA/ET signaling pathway were mainly correlated with the secretion of amino acids, benzenoids, and heterocyclic compounds (Fig. 5C and D). Previous studies have shown that amino acid metabolites, including aspartate, asparagine, glutamine, arginine, and cysteine, can act as signaling molecules that mediate the interaction of rhizosphere microbial communities (98). However, these amino acids were not significantly different in root exudates of wild-type plants and the *Def* mutant line. In addition, studies have shown that leucine has a positive interaction with B. subtilis and *B. amyloliquefaciens* (99, 100). However, our analysis showed that the correlation between leucine and *Bacillus* was not significant. This may be because previous studies mainly focused on a specific species of *Bacillus*, while our study is based on the overall change in *Bacillus* abundance in the rhizosphere, which is more complex and diverse and is affected by more factors. Heterocyclic compounds are the largest class of compounds. In this study, the three heterocyclic exudates coregulated by the SA and JA/ET signaling pathways are all nitrogen-containing heterocycles. Among them, multiple derivatives of quinoline exhibit broad-spectrum antibacterial activity (101). Generally, terpenoids secreted by roots have strong antibacterial properties (90). They can help plants better resist invasion by pathogens. For example, ursolic acid has a good inhibitory effect on Staphylococcus aureus, Escherichia coli, Pseudomonas aeruginosa, Klebsiella pneumoniae, Shigella flexneri, and other pathogenic bacteria (102, 103). Our analysis showed that several terpenoids regulated by the JA/ET signaling pathway were positively correlated with the relative abundance of several microorganisms used for the analysis (Fig. 5C and D).

Our experiment linked the biocontrol strains, rhizosphere bacterial communities, and plant pathogens, and we speculated that the biocontrol strain could control bacterial wilt disease by regulating the plant disease resistance-related signal pathway and promoting the enrichment of *Bacillus*, *Bosea*, *Ammoniphilus*, *Flexivirga*, *Brevundimonas*, *Bordetella*, and *Dyella* in the plant rhizosphere. However, in addition to the seven genera mentioned above, the application of the biocontrol strain B. cereus AR156 also led to the enrichment of 28 other genera, and their enrichment may be related to other metabolic pathways of plants or direct attraction by the metabolites of B. cereus AR156. Therefore, future research can be carried out on other metabolic pathways of plants or the biocontrol strain’s own metabolome to further explain the principle underlying the enrichment of beneficial microorganisms. In addition, the composition of soil microorganisms is complex where alongside bacteria, there are fungi, viruses, protozoa, and other organisms. The relationships among microbes are complex, and the interactions between microbes cause changes in the microbiota that further affect plant health (104). Therefore, future studies need to focus on the effect of biocontrol strains on the interaction between the original microorganisms in the soil.

### Conclusion.

**FIG 6:**
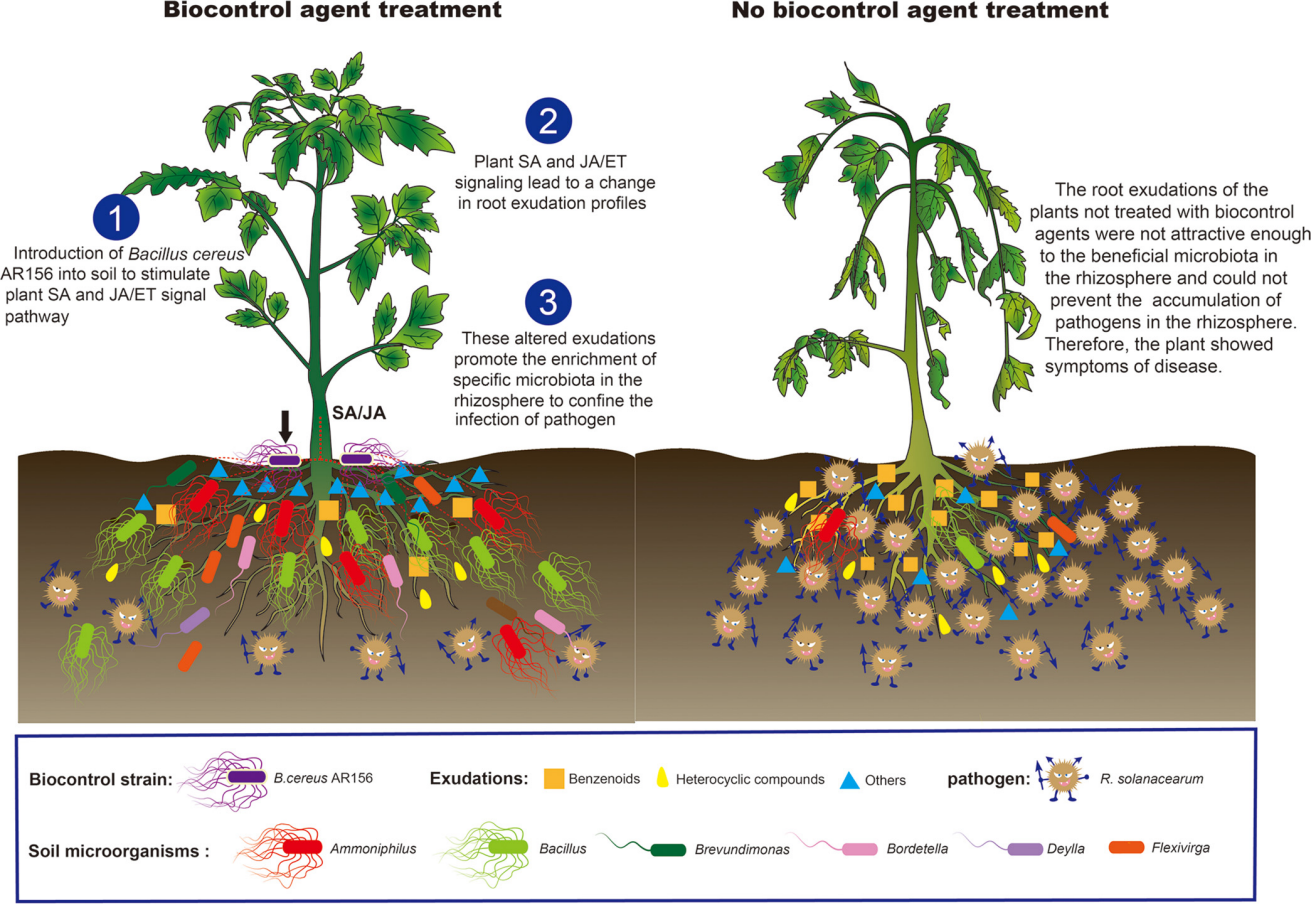
A model for tomato bacterial wilt inhibition through soil microecology amended with B. cereus AR156. Application of B. cereus AR156 to plant rhizosphere activates the SA and JA/ET signaling pathways. The activation of SA and JA/ET signaling pathways leads to a change in root exudation profiles. Changes in the root secretion spectrum promote the enrichment of disease resistance-related microorganisms in the rhizosphere to confine the infection and spread of R. solanacearum.

In this study, we found that B. cereus AR156 may promote the enrichment of beneficial microorganisms in the plant rhizosphere by regulating SA and JA/ET signaling pathways in plants, thereby playing a role in controlling bacterial wilt disease. Meanwhile, Spearman correlation analysis showed that the relative abundances of these beneficial bacteria were correlated with the secretion of root exudates. Our combined results allow us to propose a model related to the biocontrol mechanism (Fig. 6). We hypothesize that the application of biocontrol bacteria leads to the activation of plant disease resistance-related pathways. Plant resistance-related signaling leads to a change in root exudation profiles. These altered root exudates promote the enrichment of specific microbes in the rhizosphere to confine the infection and spread of pathogens. Together, the results of this study reveal a new mechanism for the SA and JA/ET signals to participate in the adjustment of plant resistance; that is, signal pathways adjust the rhizosphere microecology by changing the root exudates and thus change plant resistance. In addition, biocontrol strains can utilize this mechanism to recruit beneficial bacteria by activating disease resistance-related signaling pathways to confine the infection and spread of pathogens.

## MATERIALS AND METHODS

### Plant materials and experimental location.

The soil used in this experiment was collected at five locations of Pailou Scientific Research and Teaching Base of Nanjing Agricultural University in Nanjing, Jiangsu Province, China (32°04′15″ N, 118°76′74″ E) in August 2019. The soil was fully mixed and filtered through 20 mesh sieves for greenhouse tomato planting. The initial soil chemical properties were ammonium nitrogen, 65.17 mg/kg; pH 5.15; organic matter, 13.92 g/kg; available phosphorus, 49.02 mg/kg; and available potassium, 124.21 mg/kg.

The tomato varieties were wild-type tomato (Lycopersicon esculentum Mill. cv. Moneymaker, MM for short) and its transgenic line NahG (decreases the ability to accumulate salicylic acid); and wild-type tomato (Lycopersicon esculentum Mill. cv. Castlemart, CM for short) and its *def-1* mutant line *Def* (lacks the ability to accumulate jasmonic acid). Tomato seeds were disinfected with 10% trisodium phosphate solution, germinated in the dark, planted in the seedling tray after 3 to 4 days, and transplanted to pots after 2 weeks. Plants were grown in a 26°C greenhouse with 16-h light/8-h dark.

### Experimental design and rhizosphere sample collection.

The experimental design was divided into two parts. One part was used to study the effect of B. cereus AR156 on the composition and abundance of the bacterial community in tomato root soil. The design was as follows: the rhizosphere soil was collected 5 days after tomato transplantation and was marked as mock-0d. The remaining tomato seedlings were divided into two groups: group A, water irrigation; and group B, irrigation with a final population of 0.5 × 10^7^ CFU/mL B. cereus AR156. After 25 days of irrigation, the rhizosphere soil was collected and marked as CK-30d and AR156-30d.

The other part was used to study the differences in bacterial community composition and abundance in rhizosphere soil between the NahG transgenic line and *Def* mutant and their wild types under normal growth or pathogen infection. The soil samples were collected before transplanting and marked as C-0, D-0, M-0, and N-0, indicating the soil samples of tomato varieties on the 0th day of planting. The tomato seedlings of four lines were divided into two groups. One group was cultured after 30 days without any treatment, and the rhizosphere soil samples were recorded as C-30, D-30, M-30, and N-30. The other group was inoculated with R. solanacearum GMI1000 15 days after transplanting and then cultured for 15 days. The rhizosphere soil samples were recorded as C-R-30, D-R-30, M-R-30, and N-R-30. In the above descriptors, code C refers to wild-type tomato CM, code D refers to the *def-1* mutant line *Def*, code M refers to the wild-type tomato MM, and code N refers to the transgenic line NahG.

The collection methods of rhizosphere soil were slightly changed according to the article of Fu et al. (105). The whole tomato plant was removed from the pot, and the bulk soil and the impurities attached to the tomato root surface were removed. The plant was placed into a 500-mL centrifuge bottle containing sterile water, treated with ultrasonic waves at 70 Hz for 30 min and shaken at 28°C for 30 min. The root system was removed and centrifuged at 4°C and 10,000 rpm for 10 min, the supernatant was discarded, and the sediment was stored in a −80°C ultralow temperature freezer (105).

### Strain and culture conditions.

B. cereus was activated on LB solid medium and cultured in a 37°C incubator for 1 day. Single colonies were picked out and transferred to a glass tube containing 5 mL of LB liquid medium, shaken at 37°C and 200 rpm for 12 h and then transferred to a 1 L conical flask containing 500 mL LB liquid medium to expand the culture. R. solanacearum GMI1000/GMI1000-RFP was activated on YGPA solid medium and cultured in a 28°C incubator for 2 days. Single colonies were picked out and transferred to a glass tube containing 5 mL of YGPA liquid medium, shaken at 28°C and 200 rpm for 24 h, and transferred to a 1-L conical flask containing 500 mL YGPA liquid medium to expand the culture. The LB medium used consisted of 10 g/L tryptone, 5 g/L yeast extract, and 10 g/L NaCl (pH 7.2). The YGPA medium used consisted of 10 g/L glucose, 5 g/L peptone, and 5 g/L yeast extract (pH 7.2).

### Microbiome analysis.

Total genomic DNA was extracted from 45 rhizosphere samples (3 for each treatment) using the PowerSoil DNA isolation kit (Mo Bio Laboratories Inc., USA) according to the manufacturer’s instructions. DNA was extracted from five technical replicates per sample to minimize the DNA extraction bias. The DNA quality was assessed using a NanoDrop One spectrophotometer (Thermo Scientific, USA). The V3 + V4 regions of the bacterial 16S rRNA gene were amplified with the specific primers 341F (CCTAYGGGRBGCASCAG) and 806R (GGACTACHVGGGTWTCTAAT) with barcodes using qualified DNA as a template. After PCR amplification, bands were purified from a 1.2% agarose gel using the Gel Extraction kit (Thermo Scientific, USA). The purified amplification products (extenders) were connected to sequencing adapters, and sequencing libraries were constructed and sequenced on the Illumina platform using PE250 chemical paired-end sequencing.

After the original data were obtained by sequencing, low-quality reads were filtered using Usearch software (http://www.drive5.com/usearch/). Paired-end reads were spliced into tags, and low-quality tags were filtered. The data obtained were called clean tags. The obtained clean tags were clustered, and the chimeric tags detected in the clustering process using the Usearch software were removed, and finally the abundance and representative sequence table of operational taxonomic units (OTUs), containing 5.23 million effective reads, was obtained.

After the OTU abundance table was obtained, abundance statistics were performed based on the effective tags. According to the analysis process, species composition analysis, indicator species analysis, α-diversity analysis, β-diversity analysis, and community function prediction were performed. Redundancy analysis (RDA) correlation analysis and linear correlation analysis combined with other factors (such as environmental factors) were carried out to determine the relationships between microorganisms and the environment.

LDA score and evolutionary branching diagrams were analyzed using the “lefse” package in the python 2 environment. The β-diversity (PCoA based on Bray-Curtis dissimilarities) was calculated using the “phyloseq” package in the R environment. Welch's *t* test was used to calculate the significance of the difference between two treatments using STAMP software (106). Redundancy analysis was performed using Canoco 5 software. The species abundance stack map was plotted using GraphPad Prism 8. The linear correlation was calculated by SPSS and plotted by GraphPad Prism 8 software.

### Extraction of root exudates.

Root exudates were collected as described by Peng et al. (107), and whole seedlings were carefully removed from the pot 15 days after tomato transplantation. The adsorbed substances were rinsed from the root surface slowly with distilled water, and the root was washed three to five times carefully to ensure the integrity of the root system, and then filter paper was used to absorb the surface water. Nine seedlings of each line were randomly selected and placed in an opaque beaker containing 250 mL of distilled water. Root exudates were collected under normal photoperiod conditions. Tomato seedlings were removed after 15 days, and the root exudates were filtered with a Buchner funnel and a 0.45-μM bacterial filter, dried into powder by a freeze-drying machine, and stored at −80°C.

### Metabolome analysis.

Methanol (15 mL) was added to the lyophilized samples, and they were placed at −20°C to dissolve the samples and then sonicated with ultrasound for 10 min to fully dissolve them. liquid chromatography-tandem mass spectrometry (LC-MS/MS) technology was used for nontargeted metabolomics analysis. Positive ion and negative ion data were collected to improve the metabolite coverage using a Q Exactive high-resolution mass spectrometer (Thermo Fisher Scientific, USA). Compound Discoverer 3.0 (Thermo Fisher Scientific, USA) software was used for LC-MS/MS data processing, including peak extraction, peak alignment, and compound identification.

Spearman rank correlation coefficient was calculated by SPSS software, and a correlation heatmap was drawn by the “pheatmap” package in the R environment. The metabolite pie chart was drawn using GraphPad Prism 8 software. The mode was drawn using Adobe Illustrator software.

### Plant RNA extraction and qRT-PCR analysis.

The total RNA of plants was extracted using the TRIzol reagent according to the manufacturer’s guidelines. Real-time fluorescent quantitative PCR was performed on an ABI 7500 system using a GoTaq QPCR mix kit (Promega). *β-actin* was employed as the internal standard, the forward primer of *β*-*actin* was GGATTTGCTGGTGATGATGCT, the reverse primer of *β*-*actin* was GCATCCTTCTGTCCCAT TCC, and the amplified fragment size was 98 bp. *PR1* was tested as marker for SA signal pathway expression, the forward primer of *PR1* was AGATGTGGGTTGATGAGAAGCA, the reverse primer of *PR1* was CCTGACCCTAGCACAACCAAG, and the amplified fragment size was 128 bp. *PIN2* was tested as marker for JA/ET signal pathway expression, the forward primer of *PIN2* was GTCCGCTAAATCCCATAT, the reverse primer of *PIN2* was GATCACAATTAAAGGTGCA, and the amplified fragment size was 135 bp. The relative expression levels of genes were calculated according to the formula: ΔCT = CT^target gene^ − CT^internal standard^, amount of target gene = 2^−ΔΔCT^.

### Detection of soil physical and chemical properties.

Ammonia nitrogen (AN), available phosphorus (AP), and available potassium (AK) levels in soil were determined by a soil nutrient rapid test instrument (TPY-6A Type; Zhejiang Top Instrument Co., China). Soil total organic matter levels were determined using the high-temperature heating potassium dichromate oxidation volumetric method (108). The pH value of the soil was measured by the potentiometer method, and the water-to-soil ratio was 4:1.

### Root colonization assay.

The colonization of R. solanacearum in tomato roots was detected according to the method described by Dragoš et al. (109). The washed roots were transferred to Eppendorf tubes for weighing, and then, the obtained cell suspension was treated according to the standard ultrasonication protocol for colony counting. The experiment was conducted 7 days after the pathogen R. solanacearum GMI1000 inoculated, and each treatment had six biological repetitions.

### Electron microscopy/confocal laser scanning microscopy.

The colonization of R. solanacearum GMI1000 in roots was observed by laser confocal microscopy (Carl Zeiss; LSM780) and scanning electron microscopy (Hitachi S-3000N). The fluorescence of RFP was observed by argon laser at 561 nm, and the images were processed by ZEN 2.3 software. The scanning electron microscopy observation method was performed according to the previous description of Shi and Butenko (110). The soil on the root surface was washed with sterile water, fixed with 2.5% glutaraldehyde for more than 8 h, rinsed with phosphate buffer three times, dehydrated with an ethanol gradient for 15 min at each ethanol concentration, replaced with tert-butanol three times, and freeze dried, and the samples were then observed.

### Data availability.

The data that support the findings of this study are available in the SRA at the NCBI with the identifier PRJNA869862.
