# Supplementary material for: Plant Disease Resistance-Related Pathways Recruit Beneficial Bacteria by Remodeling Root Exudates upon Bacillus cereus AR156 Treatment
Source: Microbiol Spectr. 2023 Feb 14;11(2):e03611-22. doi: 10.1128/spectrum.03611-22 (PMC10100852; doi:10.1128/spectrum.03611-22)
Supplement: Supplemental file 8 — Fig. S1 to S7. Download spectrum.03611-22-s0008.pdf, PDF file, 1.7 MB [file spectrum.03611-22-s0008.pdf]

Supplementary Figure 1.

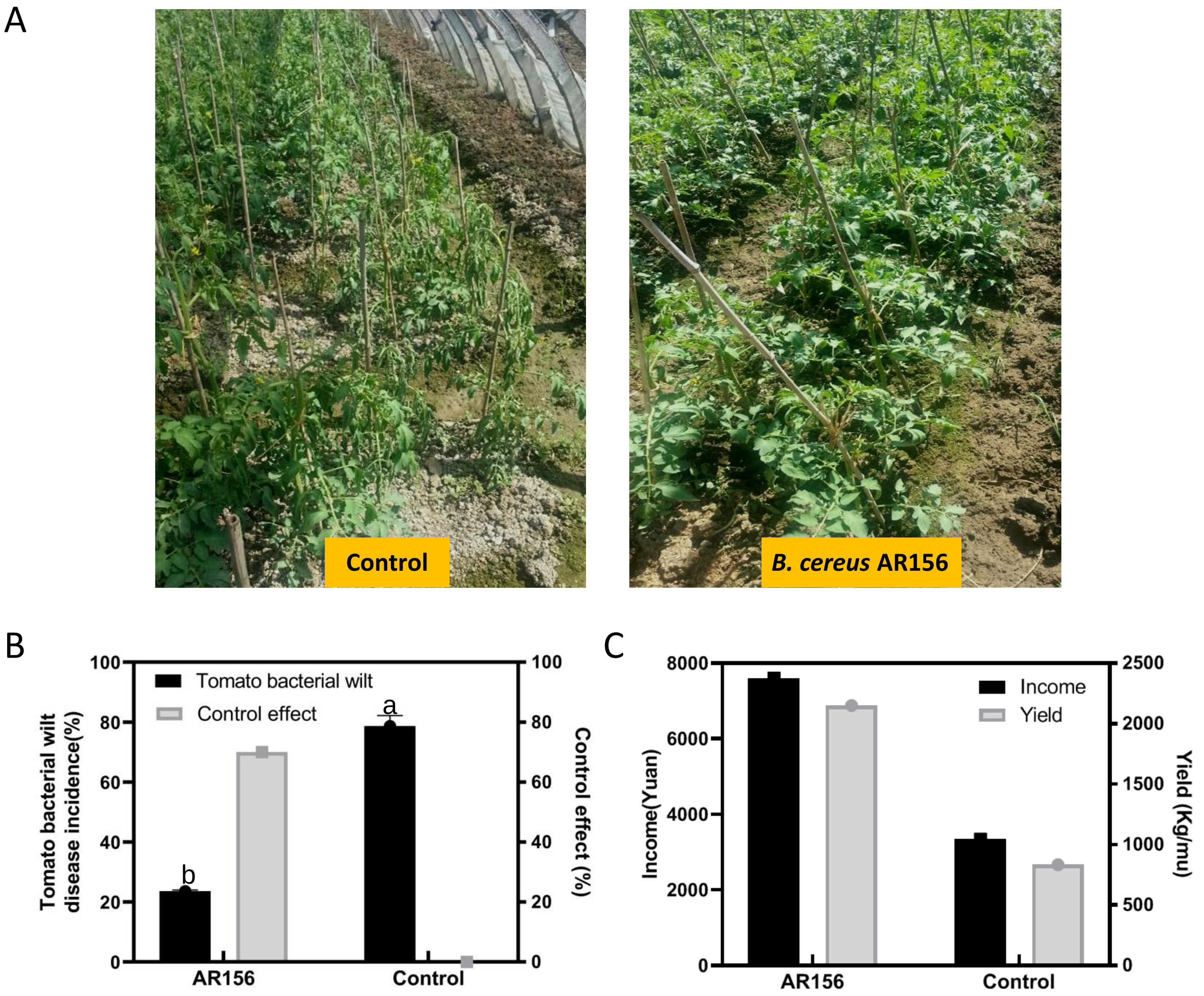

Fig. S1 *B. cereus* AR156 can effectively control tomato bacterial wilt disease in the field.

Note: **A.** The symptoms of bacterial wilt disease development on tomatoes in field. 700 liters of 20 times diluents of *B. cereus* AR156 with OD600=1.0 was used every mu of land, and it was used three times in the whole growing season of tomatoes. **B.** Incidence and control effect statistics of tomato plants with different treatments. Different lowercases meant significant difference, Duncan's new multiple range test, $P < 0.05$. **C.** Income and yield of tomato plants with different treatments.

Supplementary Figure 2

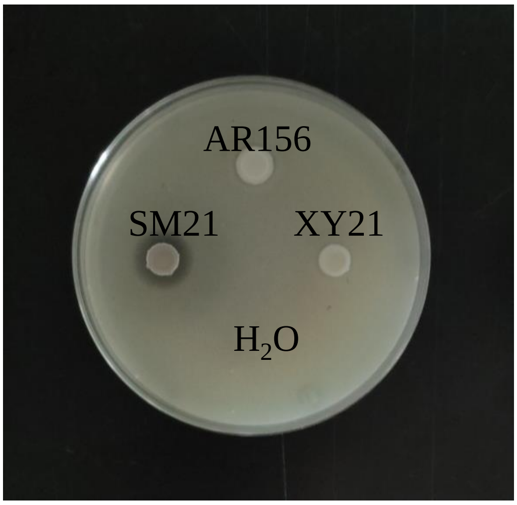

Fig. S2 ***B. cereus*** **AR156 did not directly inhibit the growth of** ***R. solanacearum*****.**

Note: SM21 as positive control; XY21 and H_2O as negative control. The test was performed on YGPA medium plate.

Supplementary Figure 3

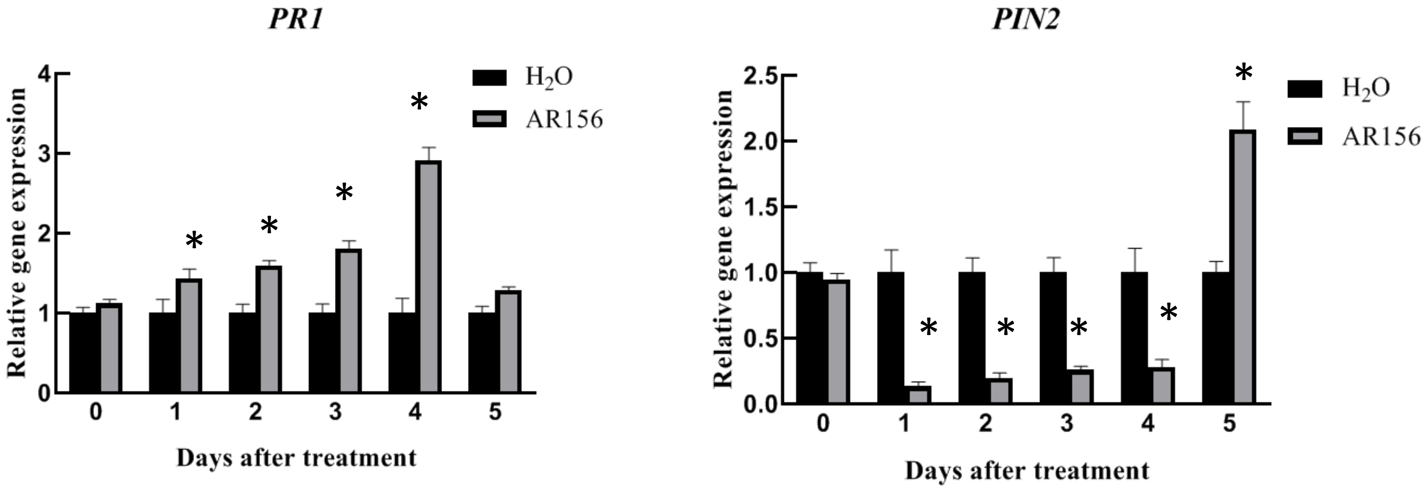

Fig. S3 ***B. cereus*** **AR156 can regulate the expression of SA and JA/ET related genes in tomato plants.**

Note: Leaves of tomatoes in different treatments were harvested at the indicated time points for extracting total RNA. Gene expression levels were determined by qRT-PCR. *PR1* gene associated with SA-mediated defense signaling pathway. *PIN2* gene associated with JA/ET-mediated defense signaling pathway. The expression values of the individual genes were normalized using *β-actin* gene as an internal standard.Data are presented as means of four replicates±SD, and error bars represent SD for four replicates. "*" meant significant difference, Duncan's new multiple range test, $P<0.05$.

Supplementary Figure 4

A

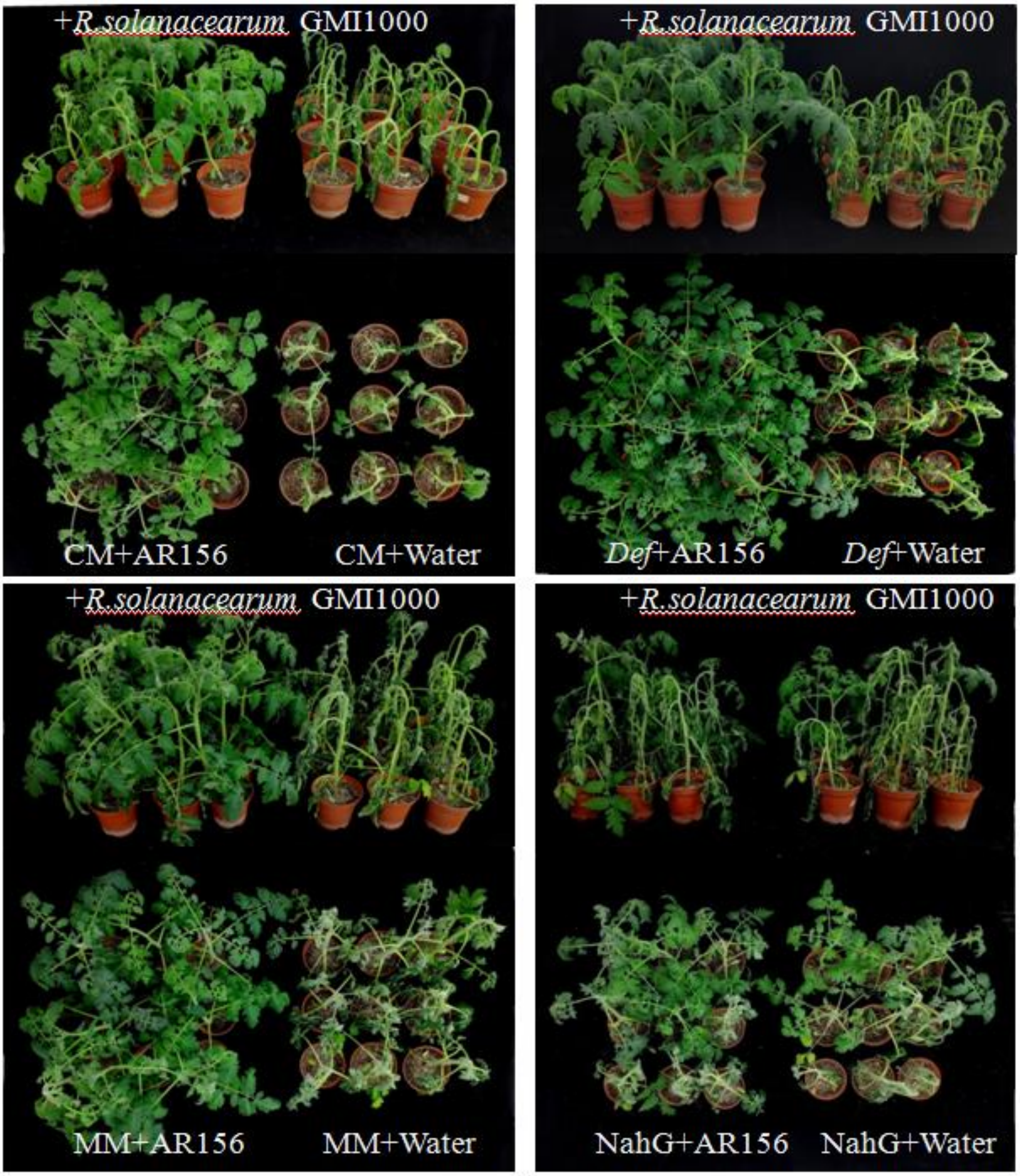

B

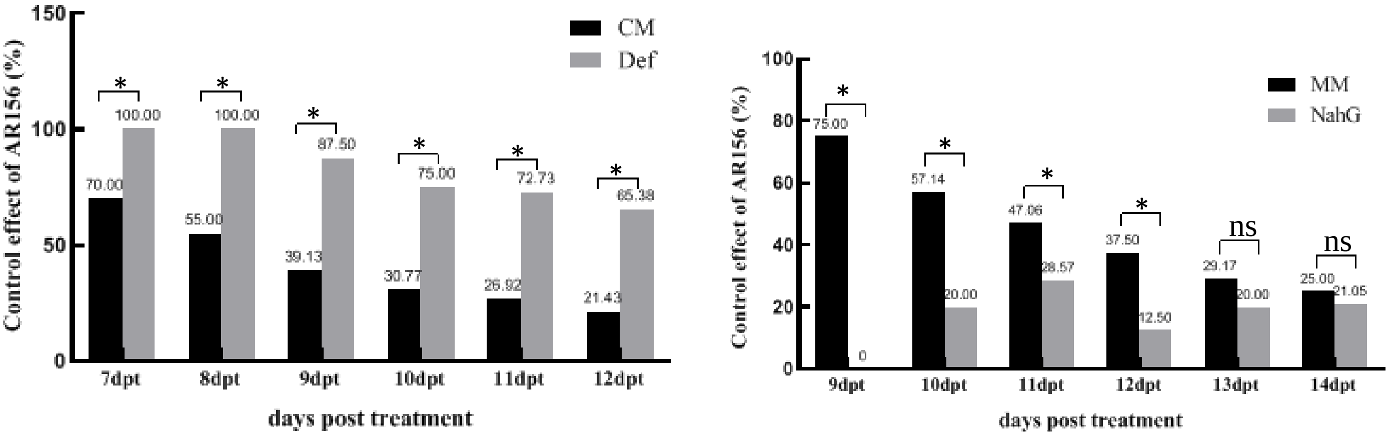

Fig. S4 The control of tomato bacterial wilt by *B. cereus* AR156 is related to SA and JA/ET signaling pathways.

Note: **A.** The symptoms of bacterial wilt disease development on tomatoes with different treatment in greenhouse experiment. The experiment was repeated 3 times, and 12 seedlings were treated each time. **B.** Control effect statistics of tomato plants with different treatments at the indicated time points. "*" meant significant difference Duncan's new multiple range test, $P < 0.05$.

Supplementary Figure 5

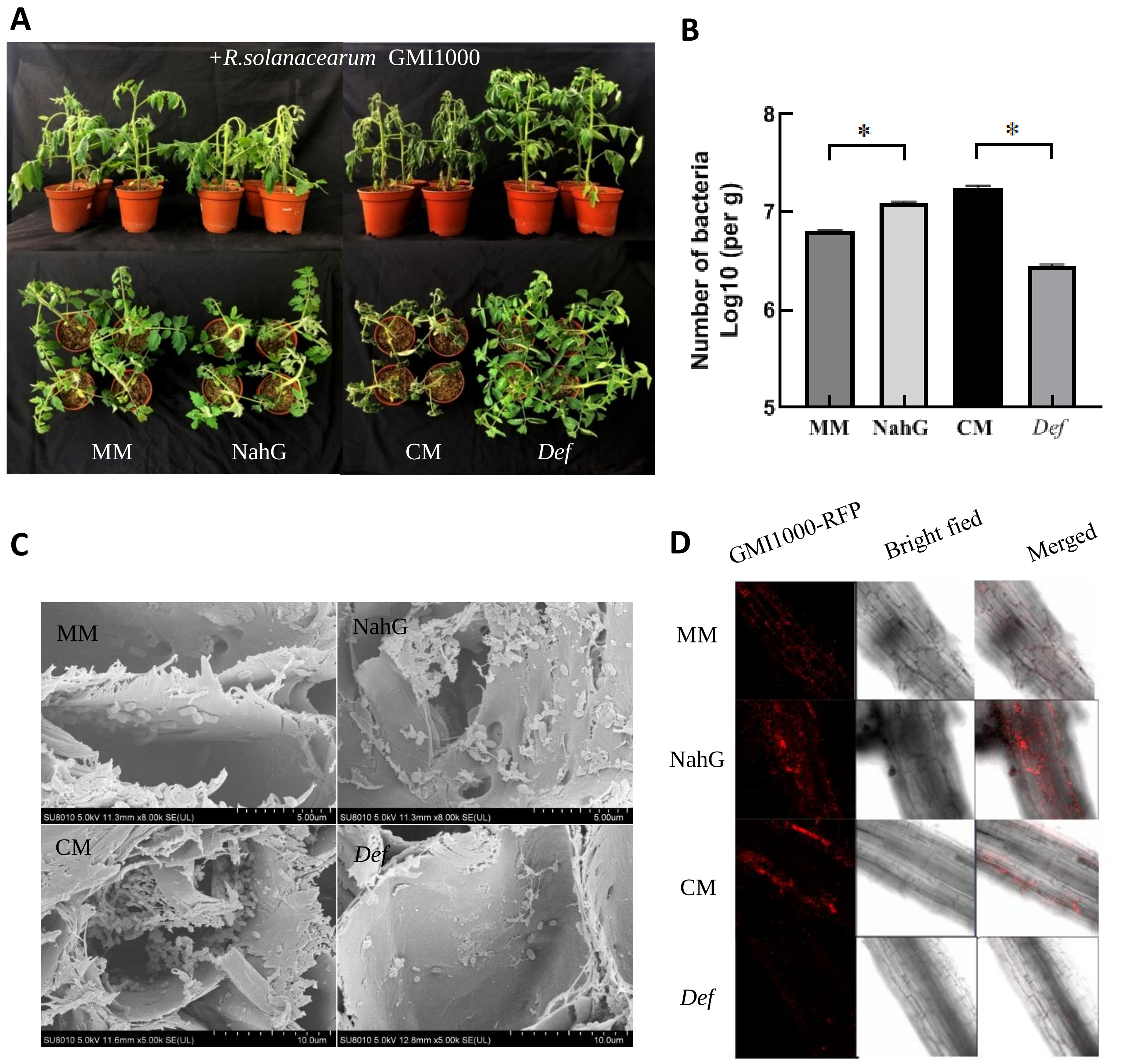

Fig. S5 The SA and JA/ET signaling pathways in tomato are involved in resistance against the bacterial pathogen *R. solanacearum* GMI1000.

Note: **A.** The symptoms of bacterial wilt disease development on tomato seedlings 10 days post *R. solanacearum* GMI1000 inoculation in greenhouse experiment. The experiment was repeated for 3 times, and 12 seedlings were treated each time. **B.** Bacteria were quantified in extracts of roots by serial dilutions at day 7 after inoculation. The asterisk indicates statistically significant differences as determined with Duncan's new multiple range test ($P < 0.05$). The experiment was repeated 3 times, and 6 seedlings were treated each time. Bars represent the average of three replicates and error bars show standard deviations. **C.** Morphological details of the colonization of pathogen were visualized by scanning electron microscopy at ×8,000(MM and NahG) and ×5,000(CM and *Def*) magnifications. **D.** The colonization of GMI1000-RFP at the root of NahG transgenic lines, Def deletion mutants, and their corresponding wild-type plants MM and CM observed by laser confocal microscopy. The experiment was repeated 3 times, and 6 seedlings were treated each time.

Supplementary Figure 6

A

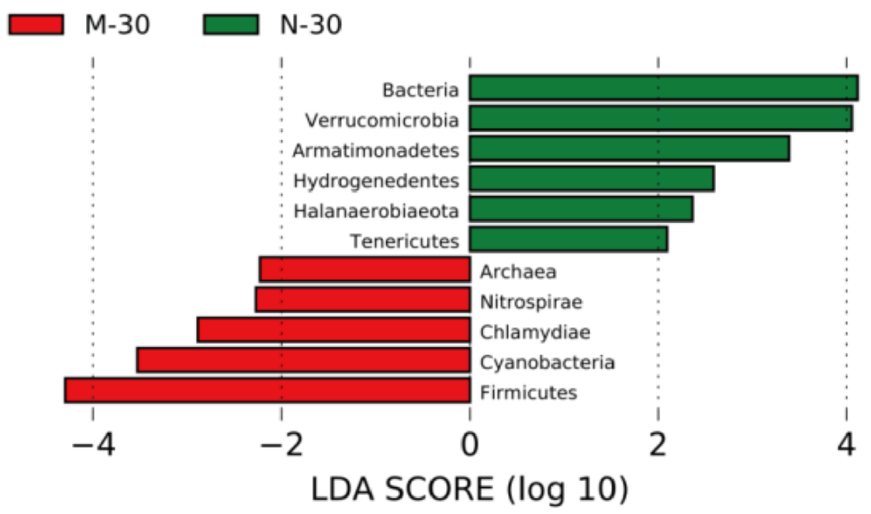

B

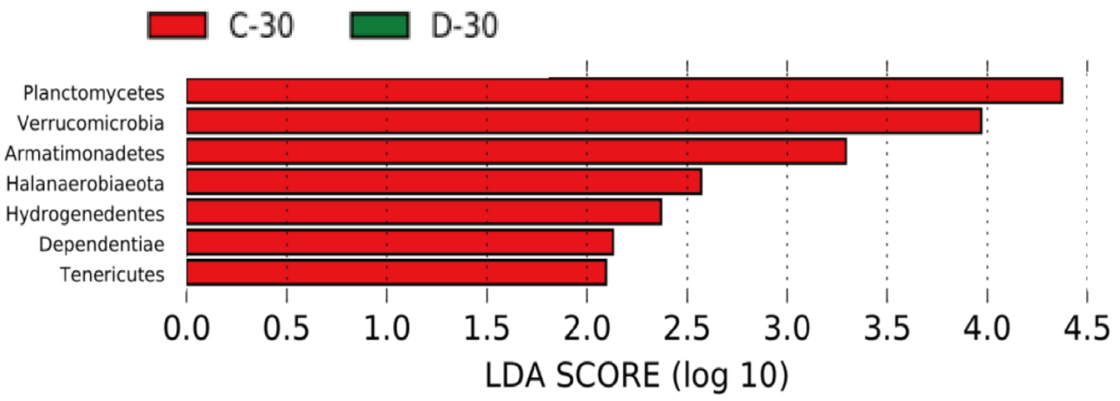

Fig. S6 Differences in rhizosphere bacterial community of wild-type and mutant line at phylum levels.

Note: **A.** Difference analysis of rhizosphere bacterial between wild-type MM and NahG transgenic line at phylum levels based on linear discriminant effect analysis (LDA>2). **B.** Difference analysis of rhizosphere bacterial between wild-type CM and *Def* deletion mutants at phylum levels based on linear discriminant effect analysis (LDA>2).

Supplementary Figure 7

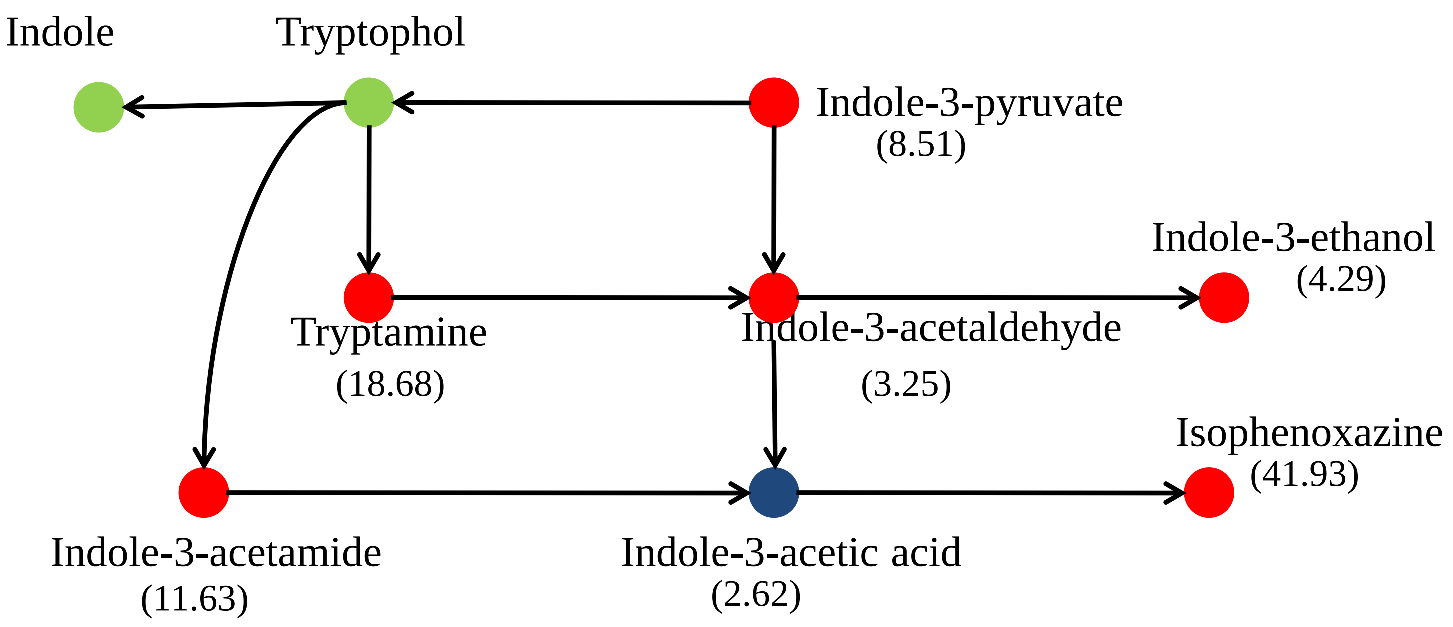

Fig. S7 Differences in compounds related to tryptophan metabolism between wild-type CM and *Def* mutant line.

Note: The green circle indicates no significant difference, the red circle indicates that the wild type is significantly higher than the mutant, the blue circle indicates that the wild type is significantly lower than the mutant, and the number in parentheses indicates the Fold-Change.
